# Meta‐analysis of Bioaccumulation Data for Nondissolvable Engineered Nanomaterials in Freshwater Aquatic Organisms

**DOI:** 10.1002/etc.5312

**Published:** 2022-03-30

**Authors:** Yuanfang Zheng, Bernd Nowack

**Affiliations:** ^1^ Technology and Society Lab Swiss Federal Laboratories for Materials Science and Technology Gallen Switzerland

**Keywords:** Meta‐analysis, Nanomaterial, Bioaccumulation, Bioconcentration, Biomagnification, Aquatic organisms

## Abstract

Understanding the bioaccumulation of engineered nanomaterials (ENMs) is essential for making regulatory decisions on potential environmental risks. Research in the field of ENM bioaccumulation has increased in recent years, but the compilation and statistical analysis of the available experimental data have not been updated. We therefore performed a meta‐analysis of the existing literature on the bioaccumulation of eight types of nondissolvable ENMs (titanium dioxide [TiO_2_], aluminum oxide [Al_2_O_3_], gold [Au], fullerene [C_60_], carbon nanotubes, iron oxide [FeO_*x*_], graphene, and polystyrene) in nonmammalian freshwater aquatic organisms across three trophic levels including phytoplankton, zooplankton, and fish. Three typical endpoints were used to assess the bioaccumulation potential: the bioconcentration factor (BCF), the bioaccumulation factor (BAF), and the biomagnification factor (BMF). Our results suggest that zooplankton has greater mean logarithmic BCF and BAF values than phytoplankton (3.31 vs. 1.42) and fish (2.04). The ENMs are biomagnified in zooplankton, with a mean BMF of 17.4, whereas trophic transfer from primary consumers (zooplankton) to secondary consumers (fish) was not observed (mean BMF of 0.13). No clear dependency was identified between the physicochemical characteristics of ENMs (e.g., primary particle size, zeta potential, or shape) and bioaccumulation, except for coated versus uncoated particles accumulated in phytoplankton. Carbonaceous ENMs were found to be more bioaccumulated than the other ENMs we considered, except for TiO_2_. A meta‐analysis of bioaccumulation data can (1) deepen the understanding of bioconcentration, bioaccumulation, and biomagnification of ENMs, (2) be used to support grouping strategies as a basis for a safer‐by‐design approach for ENMs, (3) be integrated into comprehensive hazard and risk assessments, (4) promote the standardization of testing guidelines, and (5) enhance future kinetic bioaccumulation modeling. *Environ Toxicol Chem* 2022;41:1202–1214. © 2022 The Authors. *Environmental Toxicology and Chemistry* published by Wiley Periodicals LLC on behalf of SETAC.

## Graphical abstract

Meta‐analysis of bioaccumulation of non‐dissolvable ENMs in non‐mammalian freshwater aquatic organisms across three trophic levels including phytoplankton, zooplankton and fish. Three typical endpoints were used to assess the bioaccumulation potential: the bioconcentration factor (BCF), the bioaccumulation factor (BAF) and the biomagnification factor (BMF).

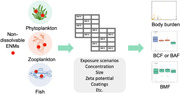

## INTRODUCTION

Engineered nanomaterials (ENMs) are manufactured for a variety of products and applications (Luoma et al., 2014). Combinations of particle size, composition, coating, shape, surface charge, or other characteristics at the nanoscale can yield distinct biological or chemical properties, quantum effects, or novel semiconductor features (Beaudrie et al., 2013).The increased production and use of ENMs results in their exposure to the environment and their interactions with ecosystems at different trophic levels (Chen et al., 2017; Johnson et al., 2017; Novak et al., 2018; Zhu et al., 2017). In Europe, approximately 9%–23% of ENMs flow to surface water, with the rest released to air (1%–19%), sludge‐treated soil (50%–60%), natural and urban soil (less than 2%), and the subsurface (10%–20%; Adam et al., 2021). Once ENMs are released into aquatic systems, they will inevitably interact with microorganisms, invertebrates, and fish, posing a potential risk to these aquatic species (Ma & Lin, 2013). In addition, ENMs could be transferred to edible tissues of fish and mollusks via the food chain, raising concerns regarding the safety of these foods (Barría et al., 2020).

Regulatory decisions on potential environmental risks are based on the extent to which compounds exhibit persistence, bioaccumulation, and toxicity (Petersen et al., 2019). This emphasizes the necessity of studying the bioaccumulation of ENMs in organisms that can then be transferred and biomagnified in the food chain. Typical endpoints used to assess the bioaccumulation potential of ENMs in aquatic organisms include the bioconcentration factor (BCF), the bioaccumulation factor (BAF), and the biomagnification factor (BMF). In general, bioconcentration is defined as the accumulation of waterborne chemicals by aquatic organisms, with contamination from food being excluded (Neely, 1980). Thus, BCF can only be measured using well‐controlled laboratory conditions. The BCF is calculated as the ratio of the concentration of the chemical in the organism or tissue (in mg/kg) at steady state to the concentration of the chemical in the surrounding medium (in mg/L; Hamelink, 1977). Bioaccumulation describes the net consequence of uptake, transformation, and elimination from all sources including respiration, contact, and ingestion, which is typically exposed to both contaminated medium and food (Neely, 1980). The BAF is calculated in the same manner as the BCF. In biodynamic studies, BCF and BAF can also be determined by the ratio of the uptake and elimination rate constants when equilibrium is not reached (Fan, Liu, Peng, et al., 2016; Hamelink, 1977; Xu & Pascoe, 1993). Biomagnification describes the relative increase in concentration from one trophic level to the next and can be ascribed to the accumulation of food (Neely, 1980). A BMF above one indicates the occurrence of biomagnification across the food chain (Utembe et al., 2018). With regard to ENMs, there are no established standards for acceptable levels of ENMs taken up by biota (Hund‐Rinke et al., 2016), but several regulatory agencies give thresholds for traditional chemicals (Arnot & Gobas, 2006). Chemicals with a BCF of 5000 or greater (log BCF of 3.7 or greater) are considered “very bioaccumulative” (European Commission, 2011; US Environmental Protection AgencyPA, 1976).

The available studies have shown that ENMs have a different bioaccumulation behavior compared with conventional substances such as hydrophobic organic chemicals. The unique characteristics of ENMs that cause ecological damage are their high specific surface area, the presence of reactive sites on the particle surface, and their tendency to translocate (Wiesner et al., 2006). Ingested ENMs accumulated in the intestine may not readily cross epithelial tissues (Edgington et al., 2014; Mao, Hu, et al., 2016; Mao, Liu, et al., 2016). It is crucial to differentiate between ingested ENMs and those that have been absorbed through epithelial tissues and have then entered the circulatory system of the organism.

Several reviews have evaluated studies about nanoparticle bioaccumulation, summarized toxicological effects of ENMs on aquatic organisms, and explained the potential uptake routes (Barría et al., 2020; Bour et al., 2015; Djearamane et al., 2016). Ma and Lin (2013) summarized ENM adsorption and internalization behavior in fish and invertebrate tissues. Vijver et al. (2018) discussed the particle size effects on bioavailability of ENMs. Petersen et al. (2019) gave suggestions on improving bioaccumulation measurements. Although these studies demonstrated ENM bioaccumulation behavior and effects, the main limitation of all of them is the lack of data‐based evaluations. A few studies have provided quantitative overviews on published experimental results. For instance, Parsai and Kumar (2020) compiled studies reporting BCF and BAF values for copper(II) oxide (CuO), zinc oxide (ZnO), and titanium dioxide (TiO_2_) nanoparticles in fish and fish organs. A centralized mesocosm database management system for ENMs (called MESOCOSM) was developed, covering silver (Ag), CuO, cerium(IV) dioxide (CeO_2_), and TiO_2_ ENMs and including bioaccumulation data (Ayadi et al., 2021; Carboni et al., 2021; Nassar et al., 2021). Bjorkland et al. (2017) provided a comprehensive review on bioaccumulation of different types of carbon nanotubes (CNTs) in plants, invertebrates, and fish by summarizing the BCF and BAF values. Utembe et al. (2018) listed the range of uptake and elimination rate constants for a number of nanomaterials, including ZnO, gold (Au), Ag, and CuO. These reviews have tended to focus on one type of organism, one type of experiment (e.g., mesocosms), or one type of ENM. Moreover, none of these reviews performed any further statistical analysis of the data.

To our knowledge, no updated meta‐analysis has been done from a statistical perspective since the review conducted by Hou et al. (2013) almost 10 years ago. These authors reviewed more than 65 papers on the bioaccumulation of ENMs in aquatic organisms under environmentally relevant exposure concentrations and conducted a quantitative comparison by using dry weight body burden. That was the first study correlating exposure concentrations and bioaccumulated concentrations in biota based on the available experimental data. Hou et al. (2013) found that ENM accumulation was low in fish (log BCF: 0.85–3.43) and high in daphnids (log BCF: 3.16–5.64). Biomagnification was not found in the food chain from daphnia to zebrafish, but from bacteria to protozoa. Moreover, their results showed a lack of dependency of BCF on the primary size, surface coating, and composition of the ENM.

In the present study, the existing scientific literature on the bioaccumulation of nondissolvable ENMs was extensively reviewed and statistically analyzed. The analysis was restricted to ENMs that do not dissolve, so that only particulate uptake played a role and not uptake of dissolved metals after partial dissolution of the ENM. There are still many challenges in monitoring the transformation between ions and nanoparticles in environmental media and the distinction between particulate and ionic metal accumulation inside organisms. Therefore, only data on bioaccumulation of nondissolvable ENMs were compiled. Nonmammalian freshwater aquatic organisms that represent three trophic levels including primary producers (phytoplankton), primary consumers (zooplankton), and secondary consumers (fish) were considered. To allow a comparison of the results of different empirical studies, a database on ENM bioaccumulation was created by grouping experiments with different exposure and elimination conditions into different scenarios. Moreover, we converted the units of body burden to the same unit (mg/kg dry wt of organisms). Key factors affecting changes in bioaccumulation were analyzed, including exposure scenario, functional feeding group, exposure concentration, type of ENM, and physicochemical characteristics of the ENMs (size, functionalization, zeta potential, and shape). Studies conducted under an environmentally relevant exposure concentration (less than 1 mg/L) were examined separately. In addition, challenges and knowledge gaps were identified. Finally, recommendations were made for experimental design and modeling.

## MATERIALS AND METHODS

### Data collection and evaluation

We collected and analyzed bioaccumulation data for eight nondissolvable ENMs (TiO_2_, aluminum dioxide [Al_2_O_3_], Au, fullerene [C_60_], CNTs, iron oxide [FeO_*x*_], graphene, polystyrene) and three organism groups representing different trophic levels (phytoplankton, zooplankton, and fish). These ENMs are usually assumed to undergo no dissolution and transformation under environmental conditions (Bjorkland et al., 2017; Brzóska et al., 2018; Di Cristo et al., 2021; Keller et al., 2021; Wohlleben et al., 2019). A systematic review and selection of the relevant literature was conducted according to Preferred Reporting Items for Systematic Reviews and Meta‐analysis (PRISMA) guidelines (Moher et al., 2010; Supporting Information, Figure S1). Specifically, a thorough literature search was performed to evaluate the bioaccumulation levels of ENMs. Relevant papers published from January 2000 to April 2021 in English were systematically searched from online public databases (PubMed, Web of Science, and Scopus) using the bioaccumulation‐related keywords listed in the Supporting Information, Table S1.

A total of 3818 records were found after the removal of duplicate papers from the three online public databases. In the selection phase, only the peer‐reviewed literature mentioning nondissolvable nanomaterials and freshwater aquatic organisms in the title or abstract were kept. Meta‐analysis and review articles were excluded. After reading through the full text, further studies were excluded. Criteria for exclusion included the inability to determine quantitative data on bioaccumulation. Data were considered relevant if at least one of the bioaccumulation endpoints (BCF, BAF, or BMF) was measured and documented, or if it could be calculated based on the given data. In addition, only adult animals were included and studies with embryos were excluded. In the last step of PRISMA, the database was complemented by identifying additional articles by analyzing the reference lists in various review articles. Finally, 98 publications were considered relevant studies, as listed in the Supporting Information, Table S6.

The database entries can be divided into six groups: (1) basic information on the publications; (2) material characterization including name, manufacturer, purity, crystal form, nominal diameter, length, coatings, shape, hydrodynamic diameter, and surface charge (zeta‐potential) in the aqueous test medium; (3) taxonomic group, full name and common name of species, and functional feeding group (only for zooplankton); (4) experimental conditions containing exposure routes, diet, exposure type, test location, illumination, and pH; (5) bioaccumulation results including nominal concentration, measured concentration, dose regime, acute or chronic studies, form measured, detection method, body burden in mg/kg organisms dry weight, endpoint (BCF, BAF, and BMF), depuration duration, depuration with food or not, body burden after depuration, and remaining accumulated percentage after depuration; and (6) tissue distribution. In addition, each study was analyzed to find information concerning (1) trophic transfer among freshwater aquatic organisms, (2) the kinetic processes involved with bioaccumulation and depuration, and (3) particle size effects on bioaccumulation. When the data points were only presented in figures in the publications, they were extracted by using WebPlotDigitizer Ver 4.2 (Rohatgi, 2021). All the collected data were compiled into a Microsoft Excel spreadsheet with three subsheets for phytoplankton, zooplankton, and fish, respectively (Supporting Information File 2).

We collected data points representing different exposure conditions, sizes, zeta potentials, or surface coatings. To minimize the over‐representation of kinetic studies reporting data under the same conditions at multiple time points, we only collected the data at the end of the uptake and elimination period. Thus, each row in the database represents an independent observation.

Because studies varied in reported units of body burden, we converted them all to mg/kg dry weight of the organisms to enable the comparison of bioaccumulation data. The conversion factor of whole‐body weight from wet to dry weight is 10 for phytoplankton (Chen et al., 2016), 12.5 for zooplankton (Tervonen et al., 2010) and five for fish (Avenant‐Oldewage & Marx, 2000). The conversion factors for different fish tissues from wet to dry are listed in the Supporting Information, Table S3. To enable consistency in data analysis, we summarized the used nanomaterial shapes into four categories, namely, spherical (alternatively, “sphere” and “particle”), sheets (for graphene), fibers (for CNTs, alternatively, “filaments”), and rods.

### Statistical analysis

To evaluate differences across multiple groups of data, we performed a one‐way analysis of variance to compute *p* values and the Tukey–Kramer post hoc approach to test the pairwise difference. When only two groups of data needed to be compared, a *t*‐test was applied. A *p* value less than 0.05 was considered statically significant (Kloke & McKean, 2014). All tests were performed using the software R Studio (R Studio Team, 2015).

### Database content

In total, 525 endpoints were collected from 98 laboratory‐based (or mesocosm‐based) studies with 38–60 entries for each endpoint, depending on how detailed the experimental conditions, the physicochemical parameters of the ENMs, and the number of tissues that were reported. Zooplankton was the most studied organism group, with 335 endpoints, followed by fish (*n* = 109) and phytoplankton (*n* = 81). Thirty percent of the relevant studies investigated trophic transfer among freshwater aquatic organisms, 46% of the relevant studies presented the kinetics of bioaccumulation, and 25% evaluated the particle size effect on bioaccumulation. More details on parameter availability are plotted in the Supporting Information, Figure S2. The number of bioaccumulation endpoints collected for phytoplankton, zooplankton, and fish against publication year was also plotted (Supporting Information, Figure S3).

## RESULTS AND DISCUSSION

### Exposure scenario

**Figure 1:**
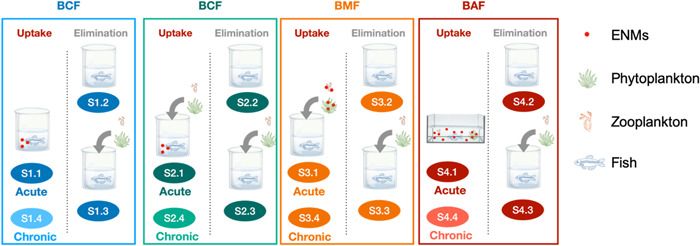
Graphical illustration of exposure scenarios regarding different uptake and elimination processes. Fish is taken as an example. Four regimes of uptake and three regimes of elimination are considered, and a distinction is also made between acute and chronic studies. The exposure duration distinguishing acute from chronic tests can be found in the Supporting Information, Table S4. S1.x, aqueous exposure only; S2.x, aqueous exposure with uncontaminated food; S3.x, dietary exposure only; S4.x, aqueous and dietary exposure; Sx.1, acute exposure with no depuration; Sx.2, depuration in clean water; Sx.3, depuration in clean water with clean food; Sx.4, chronic exposure. ENMs, engineered nanomaterials; BCF, bioconcentration factor; BAF, bioaccumulation factor; BMF, biomagnification factor.

We considered four types of uptake regimes and three types of elimination regimes, as presented in Figure 1. Uptake regimes included aqueous exposure only (S1.x), aqueous exposure with uncontaminated food (S2.x), dietary exposure only (S3.x), and aqueous and dietary exposure (S4.x). The endpoints by which bioaccumulation is quantified depends primarily on the routes of uptake. When there is aqueous exposure only (S1.x) and aqueous exposure with uncontaminated food (S2.x), BCF values are used. When organisms are exposed only to diet (S3.x), BMF values are used. In field studies, when the organisms are exposed to both contaminated water and food (S4.x), BAF values are used. In addition, BCF and BAF values were calculated based on nominal exposure concentrations. The applicability of nominal concentrations when one is determining BCF and BAF will be discussed in the *Particle concentration* section. Elimination regimes included no depuration (Sx.1), depuration in a clean water environment (Sx.2), and depuration in clean water with clean food (Sx.3). A distinction was also made between acute studies and chronic studies (Sx.4).

**Figure 2:**
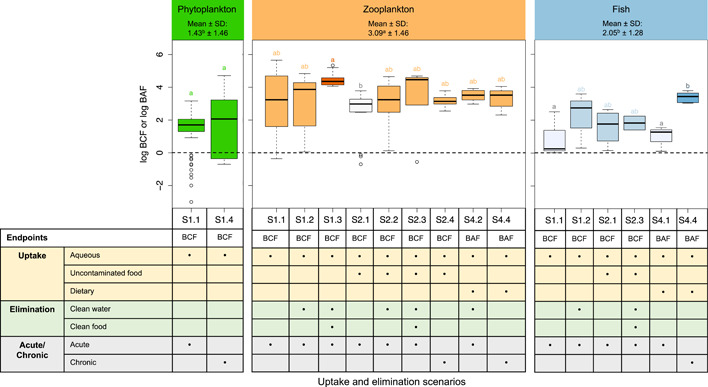
Summary of bioaccumulation results of engineered nanomaterials (ENMs) in phytoplankton, zooplankton, and fish under different uptake and elimination scenarios. A graphical illustration of the exposure scenarios is presented in Figure 1. The number of studies and data points collected for each scenario can be found in the Supporting Information, Table S2. The bioconcentration factor (BCF) and bioaccumulation factor (BAF) are given in units of L/kg dry weight of organisms. Different letters above the boxplot represent significant differences from Tukey–Kramer post hoc test or *t*‐test.

As shown in Figure 2, the mean log BCF value for zooplankton (3.09) for the chosen ENMs was greater than the one for phytoplankton (1.43) and fish (2.05). This may be due to the large gut volume of zooplankton (e.g., *Daphnia magna*) relative to the mass of the whole organism. The gut is the main organ where zooplankton accumulate ENMs (Chen et al., 2019; Fan, Liu, Xu, et al., 2016; Hu et al., 2012; Lovern et al., 2008; Petersen et al., 2009). Su et al. (2018) reported that approximately 93% of accumulated ENMs remained in the intestine. The calculated mean BMF of 17.4 presented in the Supporting Information, Table S2, indicates a high potential for trophic transfer from phytoplankton to zooplankton. However, the calculated BMF for the food chain from zooplankton to fish is well below 1, indicating that biomagnification in these food chains rarely occurs. This confirms previous findings in the literature: several studies indicated that trophic transfer from the primary producer (phytoplankton) to the primary consumer (zooplankton) occurs (Baudrimont et al., 2018; Dalai et al., 2014; Lee et al., 2015) and that trophic transfer from primary consumer (zooplankton) to secondary consumer (fish) is unlikely to occur (Dong et al., 2018; Fouqueray et al., 2013; Zhu, Wang, et al., 2010).

In addition, no significant difference was observed between acute studies and chronic studies for zooplankton. The results in Figure 2 should be interpreted with care. For example, although a significant difference between acute and chronic studies was found in fish, we should not draw any conclusion because only one study for each scenario was available to plot the figure (Supporting Information, Table S2). Another example is that the BCF values for zooplankton in the scenarios with elimination (S1.2 and S1.3) were larger than those in the scenario without elimination (S1.1). Following exposure, ENMs are able to detach from the body surface of the organism and the organism may purge ENMs from the body in a clean medium. The increase in the body burden after elimination is unexpected. The lack of a sufficient number of data points may be the reason. Therefore, we should be cautious with regard to such findings based on only a limited number of data. In our view it is not possible to distinguish between exposure scenarios in this meta‐analysis. In the later analysis on the key factors affecting bioaccumulation, the results were therefore divided into only two categories, one for dietary exposure with BMF as the endpoint and the other for all other cases with log BCF or log BAF as the endpoint.

The percentage of ENMs remaining in the organisms after elimination (remaining%) allows us to gain more insight into the elimination process of ENMs based on the body burden before and after elimination in the same study (Supporting Information, Table S2). Among all the scenarios, the average remaining percentage ranged from 11.6% to 49.5% for zooplankton and from 0% to 51% for fish. When zooplankton were depurated in clean water, 29% of the accumulated ENMs remained in their body. The addition of food facilitated depuration, and only 11.6% of the ENMs remained in their body. No data about the elimination of ENM from phytoplankton are available. Rhiem et al. (2015) provided evidence for a change in radioactivity of CNTs after transfer of exposed algae to clean water. Although the change in body burden is not available, that study offered additional support for the elimination behavior of nanoparticles attached to algae. During the uptake process, most CNTs agglomerated around cells, and single CNTs were detected in the cytoplasm, which indicated that some CNTs could transfer across cell walls. After the organisms were transferred to clean water, large amounts of ENMs detached from the cells. It should be noted that the fundamental concept of BCF and BAF is based on partitioning between water and organisms. For traditional chemicals, a partition coefficient can be derived from the ratio of the equilibrium concentrations of chemicals in two phases. However, ENMs do not reach thermodynamic equilibrium (Praetorius et al., 2014). Thus, the interpretation of BCF and BAF values for ENMs needs to consider the differences compared with the equilibrium partitioning coefficients for dissolved compounds. The values are valid only for a specific exposure system, the initial conditions, and the exposure time.

### Species‐specific factors

#### Functional feeding groups

Zooplankton can be classified into different functional feeding groups based on their feeding strategies. Four types of functional feeding groups are included in our database: filter feeders, gathering collectors, scrapers, and shredders. Filter feeders (e.g., *D. magna*) are organisms that feed by passing water through a specialized filtering feature that filters suspended matter as food. Gathering collectors catch food suspended in the water by using net‐like structures. Scrapers graze biofilm and algae that are attached to surface of rocks and plants. Shredders chew large pieces of material such as twigs and shrubs that fall into the water (Cummins, 1973; Dvořaki & Bestz, 1982).

**Figure 3:**
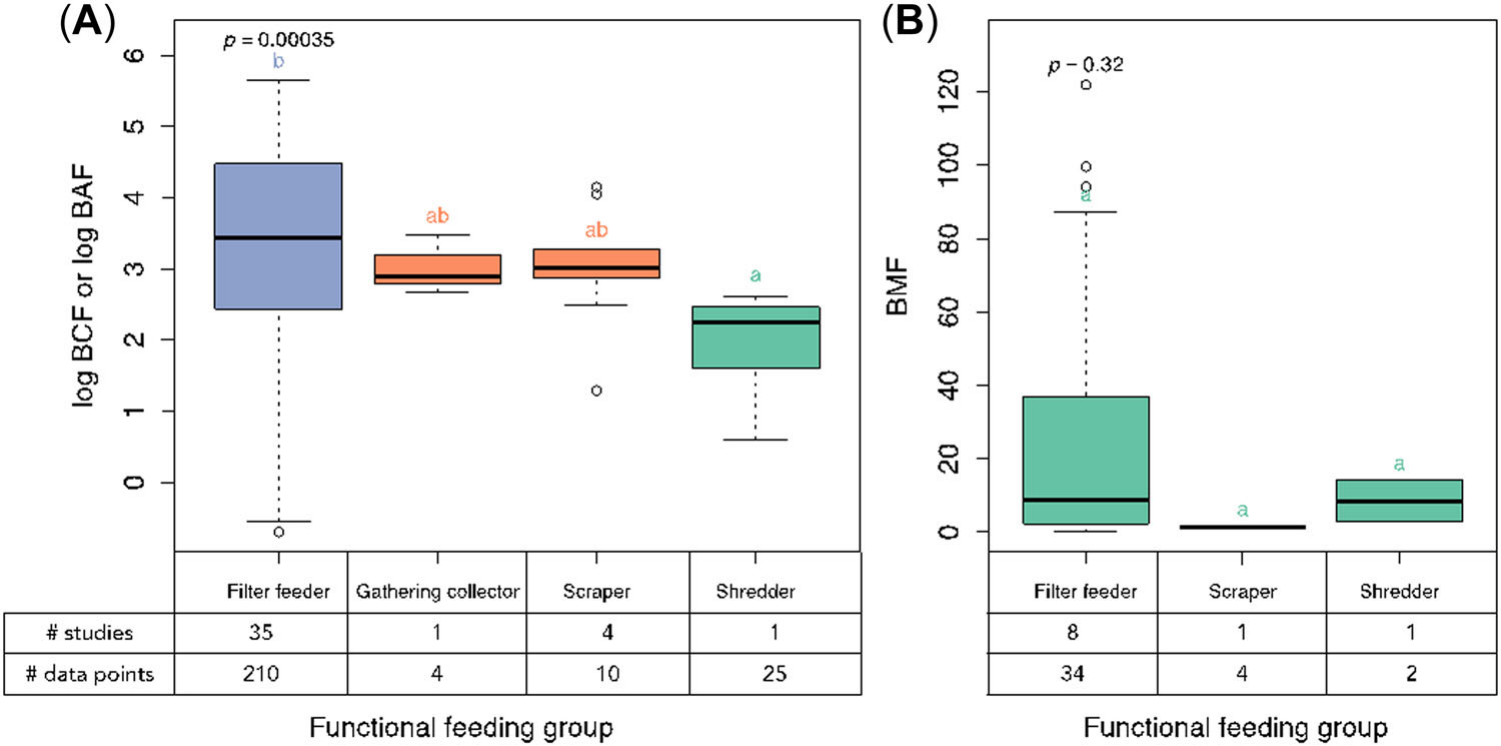
(**A**) Bioaccumulation of engineered nanomaterials (ENMs) in different functional feeding groups of zooplankton. The bioconcentration factor (BCF) and bioaccumulation factor (BAF) are given in L/kg dry weight of organisms. (**B**) Biomagnification of ENMs in different functional feeding groups of zooplankton. Different colors and letters above the boxplot represent significant differences from Tukey–Kramer post hoc test. The considered organisms in each functional feeding group can be found in the Supporting Information, Table S5.

As depicted in Figure 3, filter feeders are the most studied organisms and have the widest distribution of bioaccumulation levels. Bioconcentration factors for filter feeders range from −0.7 to 5.7 log units. Filter feeders were found to bioaccumulate ENMs more than shredders. Depending on the feeding strategies, filter feeders take up ENMs from both water and food, but shredders probably only have uptake from food ingestion. The water‐drinking behavior of filter feeders facilitates the uptake of ENMs (Rosenkranz et al., 2009). Because only one study was analyzed for each of the gathering collector, shredder, and scraper feeding groups, the results plotted in Figure 3B should not be overinterpreted. No significant differences could be observed when exposure was only through dietary uptake.

### Fish tissue distribution

**Figure 4:**
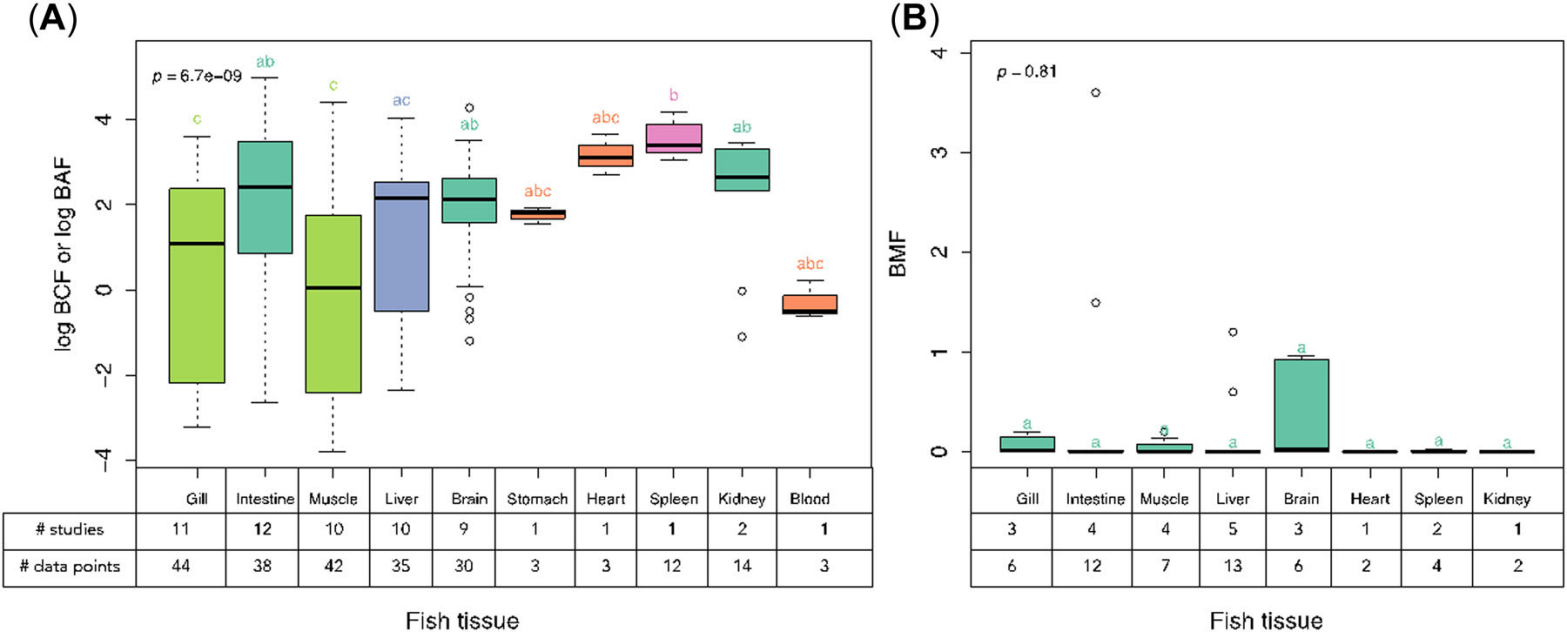
(**A**) Bioaccumulation results of engineered nanomaterials (ENMs) in fish tissues. The bioconcentration factor (BCF) and bioaccumulation factor (BAF) are given in units of L/kg dry weight of organisms. (**B**) Biomagnification level of ENMs in different fish tissues. Different colors and letters above the boxplot represent significant differences from Tukey–Kramer post hoc test.

Figure 4 details the bioaccumulation and biomagnification data for fish tissues. Among the fish tissues, intestine, gill, muscle, liver, and brain are the most studied tissues. The bioaccumulation levels of intestine, brain, spleen, and kidney were significantly higher than those of gill and muscle. In many studies, bioaccumulation of ENMs was demonstrated to be organ dependent. It has been shown that the intestine accumulates larger amounts of ENMs than other tissues (Maes et al., 2014; Perrier et al., 2018; Zhu, Carboni, et al., 2010). Some researchers have also reported that liver and kidney accumulate more ENMs than other tissues (Cunha et al., 2020; Geffroy et al., 2012; Uzo‐God et al., 2019). The reason for this is probably that liver and kidney are the organs primarily responsible for metabolism, and are responsible for the distribution of chemicals from organ to organ (Vijver et al., 2018). This is in partial agreement with our results showing that kidney accumulates more ENMs than gill and muscle, with the mean values of log BCF or log BAF at approximately 3. However, the bioaccumulation results of liver (log BCF or log BAF) had a relatively wide range, between −2 and 4. Muscle had the widest range of bioaccumulation levels, from −3.8 to 4.4 (on a log scale). Muscle has been reported to be the tissue with the lowest bioaccumulation level (Ates et al., 2016; Geffroy et al., 2012). This differs from our findings. When only dietary exposure was available, no significant difference between BMFs in fish tissues could be observed. Except for some outliers, the BMFs in all the fish tissues depicted in Figure 4B were below one, which means that biomagnification does not occur.

### Particle concentration

**Figure 5:**
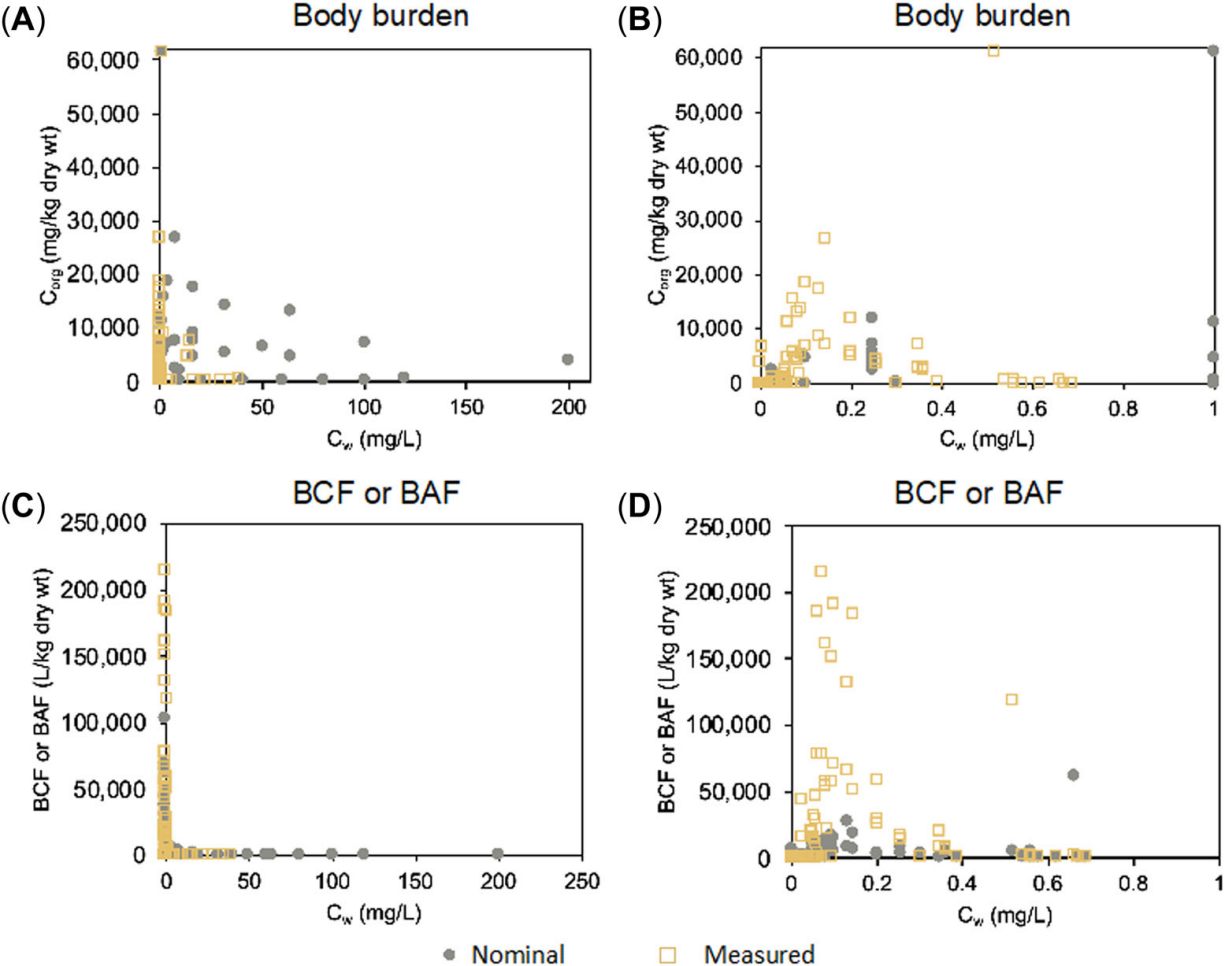
Influence of nominal concentration and measured concentration of engineered nanomaterials (ENMs) on bioaccumulation results of zooplankton. (**A**) Organism–water distribution of ENMs. C_w_ indicates the aqueous concentration of ENMs. Corg indicates body burden. (**B**) Organism–water distribution of ENMs under environmentally relevant exposure concentration (C_w_ < 1 mg/L). (**C**) Bioaccumulation endpoints (bioconcentration factor [BCF] and bioaccumulation factor [BAF]). (**D**) Bioaccumulation endpoints (BCF and BAF) under environmentally relevant aqueous concentration (C_w_ < 1 mg/L).

Figure 5 illustrates the effects of nominal and measured concentrations of particles (C_w_) on bioaccumulation and body burden (C_org_) of zooplankton. Environmentally relevant exposure concentrations (less than 1 mg/L) are shown separately. When the measured C_w_ was low, the variation in C_org_ and bioaccumulation endpoints (BCF and BAF) was substantial. As depicted in Figure 5A and C, C_org_ ranged from 0.002 to 60,000 mg/L. Bioaccumulation endpoints calculated based on measured C_w_ ranged from 6 to 214,000 L/kg. When C_w_ increased above 15 mg/L, C_org,_ and bioaccumulation endpoints decreased significantly, below 300 mg/kg and 10 L/kg, respectively.

Figure 5B and D depicts the bioaccumulation behavior of zooplankton at environmentally relevant exposure concentrations of ENMs. As the measured exposure concentration of ENMs in the medium increased, C_org_, BCF or BAF first increased and then decreased. The turning point was at an approximate concentration of 0.15 mg/L, where organisms seem to reach the maximum amount of ENMs they can take up. This turning point could be attributed to the agglomeration, aggregation, and sedimentation of ENMs at high exposure concentrations (Hudson et al., 2019).

**Figure 6:**
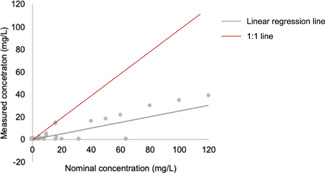
Measured concentrations of engineered nanomaterials (ENMs) in the medium against nominal concentration of ENMs added for all studies reporting both metrics.

There was no clear correlation between body burden and bioaccumulation endpoints and nominal exposure concentrations. This is in line with previous findings in the literature that there is no effect of nominal exposure concentrations on nano‐TiO_2_ uptake (Fan, Liu, Xu, et al., 2016; Federici et al., 2007). In contrast, Tao et al. (2011) reported that the body burden of C_60_ in *D. magna* increased with increasing nominal concentration. Nominal concentrations were shown to underestimate bioaccumulation levels compared with BCF or BAF, as determined from the measured concentrations shown in Figure 5D. Measured concentrations are plotted against nominal concentrations in Figure 6. The slope of this linear trend line was estimated to be far below the 1:1 line. This suggests that the fraction of ENMs present in the exposure medium can be considerably reduced due to aggregation, agglomeration, or sorption to the vessel wall under experimental conditions. As highlighted by Vijver et al. (2018), the uptake of ENMs by organisms is not a simple expression of the dose–response relationship. A steady decrease in the measured concentration in the exposure medium with time was observed (Pakrashi et al., 2013). As the concentration of ENMs increased, the bioaccumulation declined more rapidly. Therefore, the use of nominal concentrations may not be able to predict the bioavailability of ENMs in exposed organisms.

### Types of ENMs

**Figure 7:**
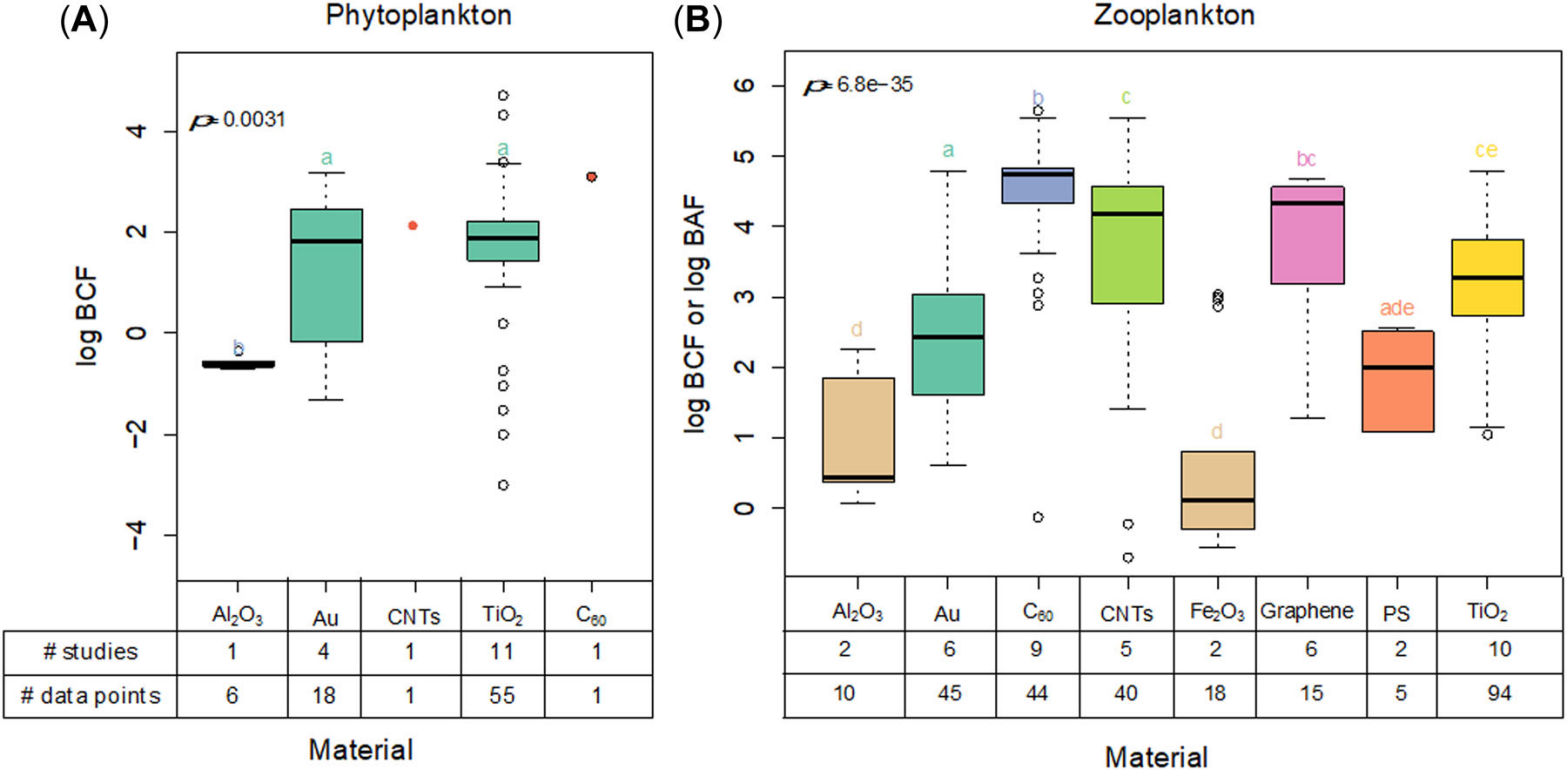
Bioaccumulation of different types of engineered nanomaterials (ENMs) in (**A**) phytoplankton and (**B**) zooplankton. Different colors and letters above the boxplot represent significant differences from Tukey–Kramer post hoc test. Al_2_O_3_, aluminum oxide; Au, gold; CNT, carbon nanotube; TiO_2_, titanium dioxide; C_60_, fullerene; Fe_2_O_3_, iron oxide; PS, polystyrene.

The bioaccumulation of different ENMs is shown in Figure 7A. Two of the most studied materials for phytoplankton are Au and TiO_2_, with a wide range of log BCF values from −3 to 4 and a similar median at approximately 2. Although Al_2_O_3_ (mean log BCF = 1.5) has a significantly lower BCF than Au and TiO_2_, this is only based on one single study. The BCFs of CNTs and C_60_, with log BCF of 1.8 and 3.1, are based on just one single data point, and thus their BCF values should not be overinterpreted.

**Figure 8:**
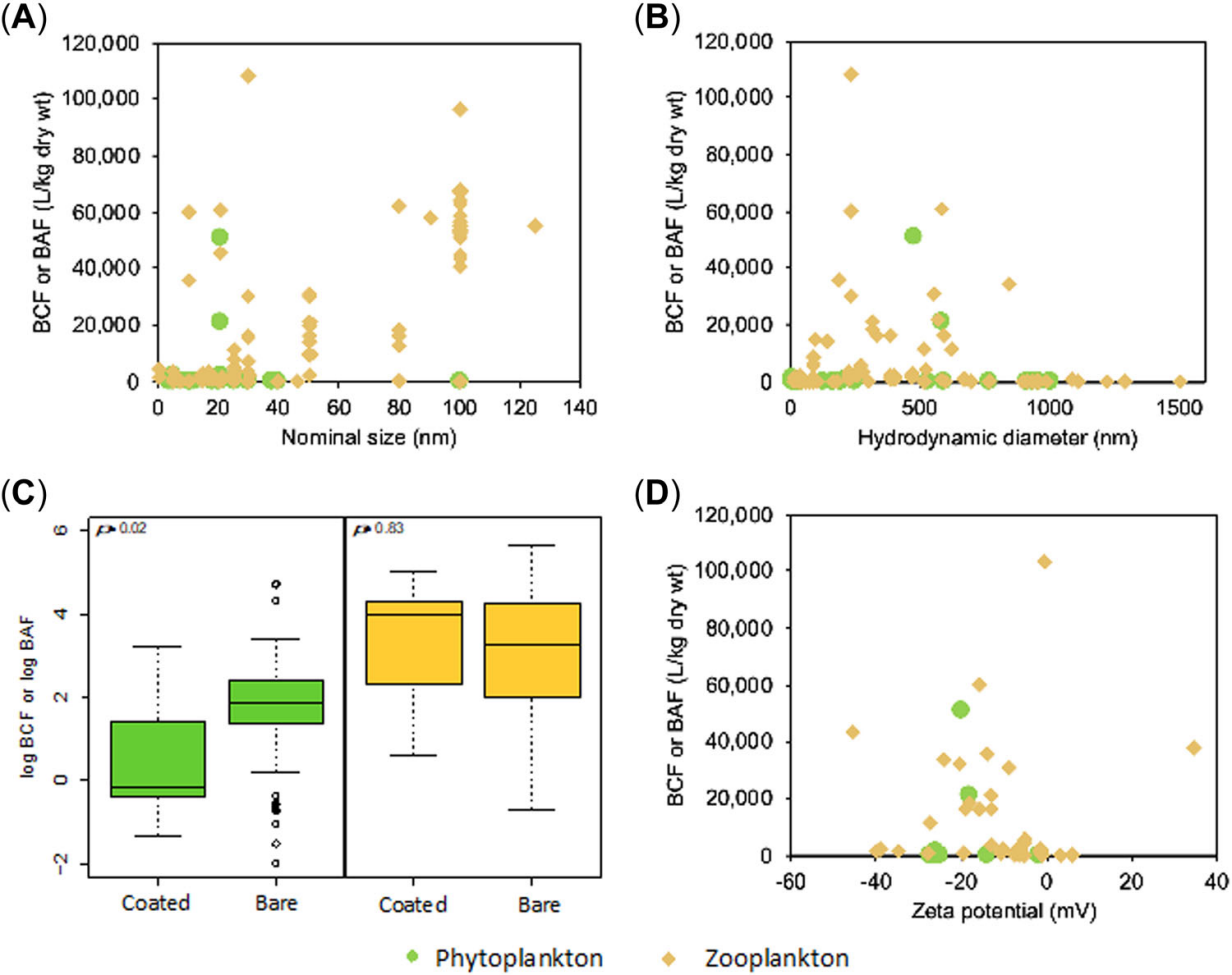
Influence of (**A**) nominal size, (**B**) hydrodynamic diameter, (**C**) coatings, and (**D**) zeta potential on bioaccumulation level in phytoplankton and zooplankton. BCF, bioconcentration factor; BAF, bioaccumulation factor.

Compared with phytoplankton, the bioaccumulation level (log BCF or log BAF) of zooplankton plotted in Figure 7B was overall higher, ranging from −1 to 5.5. The most studied nanomaterials for bioaccumulation in zooplankton are TiO_2_, Au, C_60_, and CNTs, with more than 40 data points collected for each of them. The lowest bioaccumulation in zooplankton was found for Al_2_O_3_ and Fe_2_O_3_, with log BCF or log BAF in the range of −0.6 to 3. The carbonaceous ENMs (median in a range of 4–5) were more bioaccumulative than the other ENMs (median in a range of 0–3) except for TiO_2_. The carbonaceous ENMs compiled in our database include C_60_, CNT, and graphene. We investigated the effects of hydrodynamic diameter and zeta potential on bioaccumulation for these two groups of ENMs, carbonaceous and other (except for TiO_2_). As shown in the Supporting Information, Figure S4, the hydrodynamic diameter of carbonaceous ENMs clustered predominantly between 140 and 600 nm. The other ENMs were more distributed, at sizes less than 100 nm and larger than 600 nm. When these data were compared with the whole sets of data presented in Figure 8B, they were found to be inconsistent with the findings that carbonaceous ENMs with hydrodynamic diameter values between 180 and 900 nm had a higher level of bioaccumulation. In terms of zeta potential, no difference between the two groups could be identified (Supporting Information, Figure S4). The three carbonaceous nanoparticles have very different shapes (spheres, fibers, and plates). We did not observe any significant difference in bioaccumulation among the different ENM shapes (Supporting Information, Figure S5). The higher uptake could be related to the higher hydrophobicity of the carbonaceous materials and their higher tendency to partition into a nonaqueous phase compared with metal oxides (Kidd et al., 2018).

### Physicochemical characteristics of ENMs

The influence of physicochemical characteristics on bio‐uptake is presented in Figure 8. As shown in Figure 8A, no size dependence on bioaccumulation was found using nominal particle size. Our results are consistent with some previous findings that nominal size does not influence ENM uptake into organisms (Mehennaoui et al., 2018). These authors demonstrated that the effect of the coating over‐rides the effect of the particle size. In other studies, when only one variable was examined (nominal particle size), a higher BCF was reported when zooplankton were exposed to ENMs of smaller nominal size (Chen et al., 2019; Krystek et al., 2016). Fan, Liu, Xu, et al. (2016) also noted a similar relationship at low exposure concentration (0.1 mg/L). Zhu et al. (2012) demonstrated a strong dependency of the uptake of ENMs on particle size. There are several possible explanations for the nominal size‐dependent bioaccumulation. Particles between 4 and 10 nm in size can go through the membrane bilayer by direct penetration, and the large specific surface area of small particles induces a more efficient interaction with cells (Vijver et al., 2018; Wong et al., 2010). The uptake of particles between 10 and 100 nm in size is dominated by pinocytosis. The key pathway for agglomerated and coated particles larger than 100 nm is phagocytosis (Zhu et al., 2013).

As shown in the Supporting Information, Figure S2, in all studies that reported information on the nominal size of ENMs based on transmission electron microscopy, only 50% of the studies also reported the hydrodynamic diameter in the medium. Because aquatic organisms are exposed to ENMs in an aqueous medium, the hydrodynamic diameter is the more relevant metric than the nominal size (Parsai & Kumar, 2020; Zhong et al., 2017). Figure 8B shows that the BCF or BAF values were below 20,000 L/kg, when the hydrodynamic diameter was less than 180 and larger than 900 nm. When the hydrodynamic diameter was between 180 and 900 nm, the BCF or BAF values spanned a wide range, from 0.5 to 108,000 L/kg. Owing to the high surface‐to‐volume ratio of particles with smaller nominal size, larger aggregates are formed that are less stable (Fan, Liu, Xu, et al., 2016). On the other hand, Chen et al. (2019) showed that TiO_2_ nanoparticles with different nominal sizes (5, 10, 20 and 100 nm) aggregated to similar hydrodynamic sizes of approximately 900 nm.

Figure 8C shows the effects of the particle coatings on bioaccumulation in phytoplankton and zooplankton. The bioaccumulation level of bare ENMs in phytoplankton was significantly higher compared with coated ENMs. In zooplankton, no significant difference was observed between accumulation of bare and coated ENMs. Only a few studies have compared the bioaccumulation of bare and coated particles under the same experimental conditions. Most studies examined the effects of different types of coatings on bioaccumulation, and comparisons with bare particles were lacking. Coating‐dependent uptake in crustaceans has been documented for ENMs with various coatings, including silica, alumina, citrate, mercaptoundecanoic acid, and polyethylene glycol (Fan, Liu, Xu, et al., 2016; Mehennaoui et al., 2018; Skjolding et al., 2014; Wray & Klaine, 2015). These authors argued that hydrophobic coatings promote a less stable suspension, which enhances bio‐uptake. Hydrophobic particles lead to nonspecific binding to the membrane (Teubl et al., 2015), are incorporated into the bilayer and therefore may directly penetrate the membrane. For such particles, the interior of the membrane serves as a trap, not a barrier (Marrink & Berendsen, 1996). However, hydrophilic particles only adsorb to the membrane and are not incorporated into the bilayer (Li et al., 2008). Therefore, hydrophobic particles are more likely to accumulate than hydrophilic particles.

In addition, many studies have documented that silicon dioxide (SiO_2_) and aluminum hydroxide (Al(OH)_3_) layers enhance the dispersion of nano‐TiO_2_ in an aqueous medium (Lin et al., 2002; Liu et al., 2009; Zhang et al., 2010). The zeta potential is closely related to the functionalization of ENMs. The Al(OH)_3_ coatings have been demonstrated to significantly increase the zeta potential of nano‐TiO_2_, whereas the SiO_2_ coatings did not obviously alter the zeta potential because of their lower polarity (Fan, Liu, Xu, et al., 2016). The absolute values of the zeta potential of ENMs were smaller than 30 mV, indicating that these suspensions were not under stable conditions and tended to aggregate. Figure 8D shows no clear correlation between zeta potential and bioaccumulation in phytoplankton and zooplankton. In addition, Supporting Information, Figure S5 reveals no significant difference in bioaccumulation among different shapes (spheres, fibers, sheets, or rods) of ENMs for both phytoplankton and zooplankton.

## CONCLUSIONS AND OUTLOOK

The present study has enabled a comparison of the bioaccumulation of eight types of nondissolvable ENMs across three trophic levels of aquatic organisms and provides valuable input for the environmental risk assessment of ENMs. In terms of food safety, biomagnification results so far suggest only a low probability of ENM transfer up the food chain from zooplankton to fish. The bioaccumulation level in edible parts of fish (muscles) was low, with a median log BAF value of approximately 0. No study compiled in our database showed evidence of high ENM transport across the gut tract. Thus, nondissolvable ENMs seem to pose a negligible food safety threat to humans through the pathway of fish consumption.

We explored the relationship between the bioaccumulation of ENMs and several extrinsic factors (exposure scenarios and concentrations) and intrinsic factors (physicochemical properties of ENMs). Based on the current knowledge, the different uptake and elimination scenarios did not affect the bioaccumulation of ENMs in aquatic organisms. The results of the meta‐analysis showed no significant difference between the mean values of bioaccumulation endpoints from acute and chronic studies, with and without food. In addition, no clear dependency was found between some of the physicochemical characteristics (nominal size, zeta potential, and shape) of ENMs and bioaccumulation.

The identity of ENMs was found to influence the bioaccumulation in zooplankton to some extent. Carbonaceous ENMs were found to be more bioaccumulated than the other ENMs we considered, except for TiO_2_. However, this may be an indirect effect of different particle sizes of the two groups because we found that the hydrodynamic diameter of the carbonaceous ENMs was in the range of 140–600 nm, where higher bioaccumulation level was observed, whereas the hydrodynamic diameter of other ENMs clustered outside this range, where the bioaccumulation was lower. We further observed that surface treatment plays a role in the bioaccumulation in phytoplankton because bare ENMs accumulated more in the organisms than coated ENMs. Moreover, our results imply that the use of nominal concentrations would underestimate the actual level of bioaccumulation, especially under environmentally relevant aqueous concentrations (less than 1 mg/L). This clearly suggests that future empirical research should report both nominal and measured concentrations of ENMs in the medium during exposure.

Our database revealed a few gaps in the current knowledge of ENM bioaccumulation, which were also reported in other reviews (Comandella et al., 2020; Juganson et al., 2015). The physicochemical properties of ENMs in many cases are not reported sufficiently, especially characterization of the ENM in the aqueous test medium during exposure, such as the hydrodynamic diameter or the zeta potential. Such information is critical to correlate the physical and chemical alterations of ENMs in the ecosystems to a biological activity. In addition, relatively few experiments have been conducted with phytoplankton and fish. Therefore, we can hardly draw any conclusions on the effects of the physicochemical properties of ENMs on phytoplankton and fish. More functional feeding groups of zooplankton other than filter feeders should also be investigated, including gathering collectors and shredders. Very few data were available in our compiled database on the bioaccumulation level in some fish tissues, such as stomach, heart, spleen, kidney, and blood. Such information could be relevant to enable the development of physiologically based pharmacokinetic modeling (PBPK) in the future. For PBPK modeling, a larger number of different fish species with different physiologies would be needed. In addition, our database identified that so far only a few studies have documented uptake and elimination kinetics. Such data could also help to pave the way for the development of kinetic bioaccumulation models.

To better understand the actual bioaccumulation behavior of ENMs, more environmentally relevant exposure scenarios via the use of different exposure methods and systems are needed, especially employing mesocosm and field studies (Ayadi et al., 2021; Nassar et al., 2021). This information will help in better understanding the effect of processes like aggregation and agglomeration on bioaccumulation and will facilitate biodistribution modeling. The findings of our meta‐analysis could be used to support the development of grouping strategies based on bioaccumulation as a basis for a safer‐by‐design approach toward ENMs (Jeliazkova et al., 2021; Stone et al., 2020; Wohlleben et al., 2019). Further research could be done to extend our database to the dissolvable ENMs and soil organisms.

## Supporting Information

The Supporting Information is available on the Wiley Online Library at https://doi.org/10.1002/etc.5312.

## Author Contributions Statement

**Yuanfang Zheng**: Conceptualization; Methodology; Software; Data curation; Visualization; Writing—original draft preparation. **Bernd Nowack**: Conceptualization; Supervision; Writing—review and editing; Project administration.

## Supporting information

This article includes online‐only Supporting Information.

Supporting information.

Supporting information.

## Data Availability

All data and metadata are contained in the database in the Supporting Information File 2. Data, associated metadata, and calculation tools are also available from the corresponding author (nowack@empa.ch).
