# Supplementary material for: Meta‐analysis of Bioaccumulation Data for Nondissolvable Engineered Nanomaterials in Freshwater Aquatic Organisms
Source: Environ Toxicol Chem. 2022 Mar 30;41(5):1202–14. doi: 10.1002/etc.5312 (PMC9314877; doi:10.1002/etc.5312)
Supplement: Supplementary file 1 — Supporting information. [file ETC-41-1202-s001.pdf]

Meta-analysis of Bioaccumulation Data for Non-dissolvable Engineered Nanomaterials in Freshwater Aquatic Organisms

Supporting Information

Number of pages in supporting information: 11

Number of figures: 5

Number of tables: 6

Table S1: List of keywords used in the literature search

**General Keywords**	Nano* AND (aquatic OR freshwater OR *water* OR estuarine)
**Specific Keywords**	NOT (marine OR seawater)<br>AND<br>(bio* OR *accumulat* OR *uptake OR depuration OR elimination OR *distribution OR bioavailability OR exposure OR BCF OR BCFD OR BAF OR BMF) OR ("trophic transfer" OR "food web") OR "OECD 317")
**Nanoparticles**	AND (TiO2 OR "titanium dioxide") OR (SiO2 OR "silicon dioxide") OR (Fe2O3 OR "iron oxide" OR "Fe(2)O(3)") OR (*C60 OR fullerene) OR (*CNT* OR "carbon nanotube*" OR MWNT* OR SWNT*) OR ("polystyrene" OR "PS") OR (Al2O3 OR "aluminum oxide" OR "Al(2)O(3)") OR (Au OR gold)
**Phytoplankton**	AND<br>(alga OR algae OR microalga OR "green alga" OR diatoms OR "raphidocelis subcapitata" OR "navicula pelliculosa"OR "chlorella vulgaris"OR "Anabaena flos-aquae"OR "Synechococcus leopoliensis"OR "Chlamydomonas reinhardtii"OR "Chlorella pyrenoidosa" OR "Chlorella sp." OR "desmodesmus sub-spicatus"OR "Ochromonas danica" OR "pseudokirchneriella subcapitata"OR "Scenedesmus bijugus" OR "Scenedesmus rubescens" OR "Phaeodactylum tricornutum" OR "Chlorella vulgaris" OR "OECD 201")
**Zooplankton**	AND<br>("Aquatic invertebrate" OR crustacean OR corophium OR "americamysis bahia" OR ("Asellus intermedius" OR "aquatic sowbug") OR ("Atyaephyra desmarestii" OR "Freshwater Shrimp" OR shrimp) OR ("Bryocamptus zschokkei" OR "Harpacticoid Copepod") OR ("Caecidotea sp." OR isopod) OR ("Cambarus diogenes" OR "Devil Crayfish") OR ("Caridina nilotica" OR shrimp) OR ("Ceriodaphnia affinis" OR "water flea") OR "Ceriodaphnia dubia" OR "Ceriodaphnia reticulata" OR ("Chasmagnathus granulata" OR crab) OR ("Cherax tenuimanus" OR marron) OR "Chydorus sphaericus" OR ("Cyclops viridis" OR "Cyclopoid Copepod") OR ("Cypris sp." OR ostracod) OR daphnid* OR daphnia* OR ("Daphnia magna" OR "water flea") OR "Daphnia pulex" OR "Daphnia similis" OR ("Diacypris compacta" OR ostracod) OR ("Echinogammarus berilloni" OR scud) OR "Echinogammarus meridionalis" OR "Gammarus fasciatus" OR "Gammarus fossarum" OR "Gammarus italicus" OR "Gammarus pulex" OR ("Heliodiaptomus viduus" OR "Calanoid Copepod") OR "Hyalella azteca" OR "Hyalella curvispina" OR "Lirceus alabamae" OR schrimp OR prawn OR copepod OR ("Moina macrocopa") OR ("Orconectes immunis" OR crayfish) OR ("Parastenocaris germanica" OR "Groundwater Copepod") OR ("Paratya compressa ssp. improvisa") OR ("Stenocypris major" OR "Seed Shrimp") OR ("Stenocypris malcolmsoni" OR ostracod) OR "Streptocephalus rubricaudatus" OR "Streptocephalus texanus" OR "Thamnocephalus platyurus" OR "Protozoa*" OR "Hydra" OR "Ciliate" OR "Rotifer" OR "corophium volutator" OR "Lymnaea stagnalis" OR "Potamopyrgus antipodarum" OR "Gammarus fossarum" OR "Lumbriculus variegatus" OR "OECD 202" OR "OECD 211")
**Fish**	AND<br>(Aquatic vertebrate OR Fish OR ("Acrossocheilus paradoxus" OR minnow) OR ("Ambassis sp." OR "Chanda Perch") OR ("Anguilla anguilla" OR "Common Eel") OR ("Anguilla japonica" OR "Japanese Eel") OR ("Barbus conchonius" OR "Rosy Barb") OR ("Barbus javanicus" OR Barb) OR ("Barbus ticto" OR "Two-Spot" Or "Tic Tac Toe Barb") OR ("Carassius auratus" OR "Goldfish") OR ("Catostomus commersoni" OR "White Sucker") OR ("Catostomus latipinnis" OR "Flannelmouth Sucker") OR ("Channa marulius" OR

	"Snake-Head Catfish") OR ("Channa punctata") OR ("Chanos chanos" OR "Milkfish" OR "Salmon-Herring") OR ("Cichlasoma facetum" OR "Cichlid") OR ("Cirrhinus mrigala" OR carp OR "Hawk Fish") OR ("Clarias gariepinus" OR "Zambezi Barbel") OR ("Clarias lazera" OR Catfish) OR ("Cnesterodon decemmaculatus" OR "Ten-Spotted Livebearer") OR ("Cottus bairdi" OR "Mottled Sculpin") OR ("Craterocephalus stercusmuscarum" OR "Fly Specked Hardyhead") OR ("Cyprinus carpio" OR "Common Carp" OR carp) OR ("danio rerio" OR "Zebra Danio" OR zebrafish) OR ("Etroplus maculatus" OR "Pearlspot") OR ("Fundulus heteroclitus" OR Mummichog) OR ("Galaxias maculatus" OR "Common Jollytail") OR ("Gambusia affinis" OR "Western Mosquitofish" OR "Mosquito fish") OR ("gasterosteus aculeatus" OR "three-spined stickleback" OR stickleback*) OR ("Gila elegans" OR Bonytail) OR ("Heteropneustes fossilis" OR "Indian Catfish") OR ("Ictalurus punctatus" OR "Channel Catfish") OR ("Jordanella floridae" OR Flagfish) OR ("Labeo rohita" OR Rohu) OR ("Lepidocephalichthys guntea" OR "Guntea Loach") OR ("Lepomis macrochirus" OR Bluegill sunfish)OR ("Leporinus obtusidens" OR Characin) OR ("Leuciscus idus" OR "golden orf") OR ("Melanotaenia nigrans" OR "Australian Redtailed Rainbowfi") OR ("Melanotaenia splendida ssp. inornata" OR "Checkered Rainbow Fish") OR ("Micropterus salmoides" OR "Largemouth Bass") OR ("Morone saxatilis" OR "Striped Bass") OR ("Noemacheilus montanus" OR Loach) OR ("Notopterus notopterus" OR "Asiatic Knifefish") OR ("Nuria danrica" OR Channelfish) OR ("Oncorhynchus clarkii" OR "Cutthroat Trout") OR ("Oncorhynchus clarkii ssp. pleuriticus" OR "Colorado River Cutthroat Trout") OR ("Oncorhynchus kisutch" OR "Silver Salmon") OR ("Oncorhynchus mykiss" OR "Rainbow Trout") OR ("Oncorhynchus tshawytscha" OR "Chinook Salmon") OR ("Oreochromis mossambicus" OR "Mozambique Tilapia") OR ("Oreochromis niloticus" OR "Nile Tilapia") OR ("Oryzias latipes" OR "Japanese Medaka") OR "Perca fluviatilis" OR ("pimephales promelas" OR "Fathead Minnow")OR ("Platygobio gracilis" OR "Flathead Chub") OR ("Poecilia reticulata" OR Guppy) OR ("Poecilia vivipara" OR Guaru) OR ("Prosopium williamsoni" OR "Mountain Whitefish") OR ("Pseudambassis ranga" OR "Indian Freshwater Perch") OR ("Ptychocheilus lucius" OR "Colorado Squawfish") OR ("Ptychocheilus oregonensis" OR "Northern Squawfish") OR ("Puntius sophore" OR "Pool Barb") OR ("Rasbora daniconius neilgeriensis" OR "Rasbora") OR ("Rasbora heteromorpha" OR Harlequinfish OR "Red Rasbora") OR ("Rhinichthys cataractae" OR "Longnose Dace") OR ("Salmo salar" OR "Atlantic Salmon") OR ("Salmo trutta" OR "Brown Trout") OR ("Thymallus arcticus" OR "Arctic Grayling") OR ("Tilapia zillii" OR Tilapia) OR ("Xyrauchen texanus" OR "Razorback Sucker") OR "OECD 305" OR "OECD 157" OR "OECD 203")

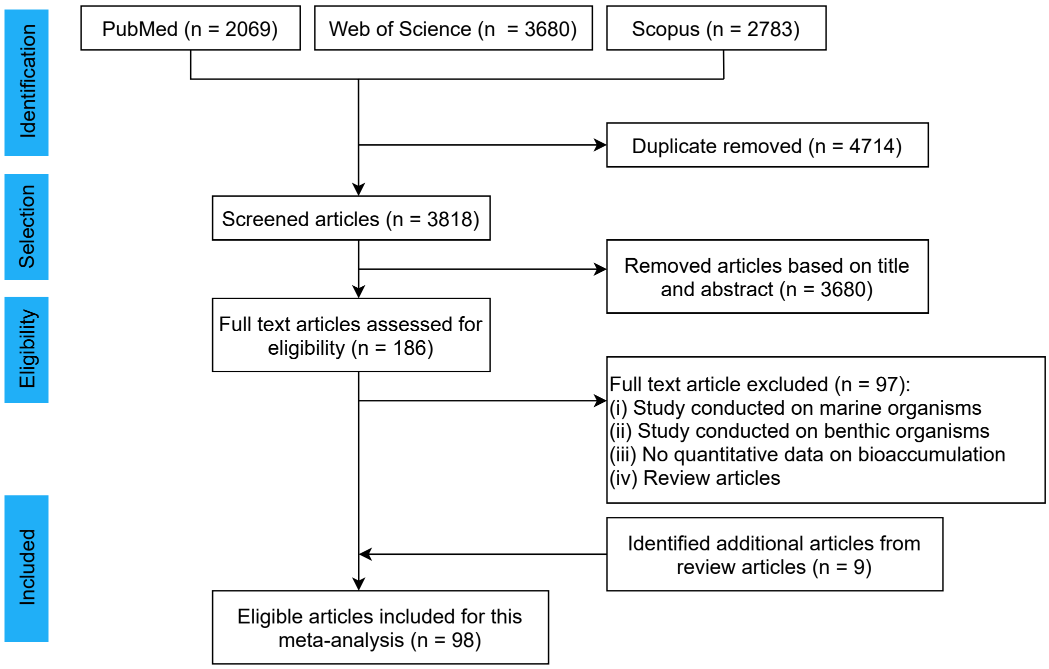

Figure S1: PRISMA flow diagram providing the different phases of data collection for the systematic review of bioaccumulation of nanomaterials in freshwater aquatic organisms.

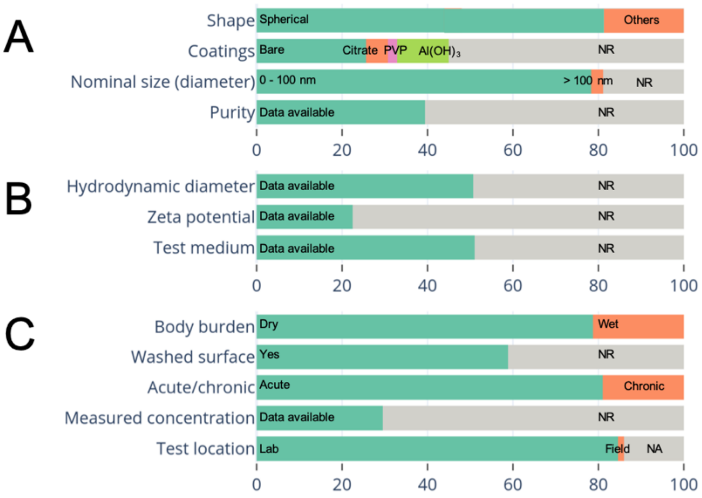

Figure S2: Availability of parameters for all the data points collected for the meta-analysis, expressed in percentage, including (A) pristine material characterization, (B) characterization in exposure medium and (C) experimental design. NR: not reported.

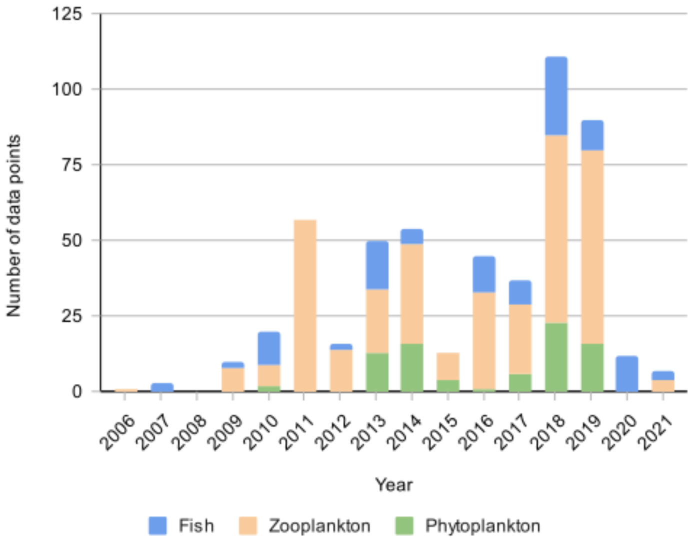

Figure S3: Number of bioaccumulation endpoints compiled in the database by publication year, for fish, zooplankton and phytoplankton respectively.

Table S2: Summary of bioaccumulation and biomagnification results of ENMs under different uptake and elimination scenarios. BCF and BAF are determined in the unit of L/kg dry weight of organisms. “Remaining%” stands for the percentage of ENMs that remains in the organisms after elimination. The exposure duration distinguishing acute from chronic tests can be found in Table S4. In the rows between lines, different letters beside the data represent significant differences. The last column takes the average per organism group. NA: not applicable. SD: standard deviation. S: number of studies. N: number of data points.

Organisms	Scenario	Uptake	Elimination/depuration	Acute/ chronic	Endpoint	S	N	Range	Mean ± SD	Remaining%	Range	Mean ± SD
Phytoplankton	S1.1	Aqueous	NA	Acute	log(BCF)	8	59	(-3.00, 3.16)	$1.28^{a} \pm 1.33$	NA	(-3.00, 4.71)	$1.43^{b} \pm 1.46$
	S1.4	Aqueous	Aqueous	Chronic	log(BCF)	10	51	(-0.70, 4.71)	$1.79^{a} \pm 1.77$	NA		
Zooplankton	S1.1	Aqueous	NA	Acute	log(BCF)	12	127	(-0.36, 5.65)	$2.94^{ab} \pm 1.72$	NA		
	S1.2	Aqueous	Clean water	Acute	log(BCF)	12	64	(0.06, 4.84)	$3.02^{ab} \pm 1.53$	29.0%		
	S1.3	Aqueous	Clean water and food	Acute	log(BCF)	2	10	(4.06, 5.20)	$4.48^{a} \pm 0.45$	11.6%		
	S2.1	Aqueous with uncontaminated food	NA	Acute	log(BCF)	4	7	(-0.70, 3.14)	$1.40^{b} \pm 1.66$	NA		
	S2.2	Aqueous with uncontaminated food	Clean water	Acute	log(BCF)	3	11	(0.12, 4.65)	$3.09^{ab} \pm 1.36$	49.5%	(-0.70, 5.65)	$3.09^{a} \pm 1.50$
	S2.3	Aqueous with uncontaminated food	Clean water and food	Acute	log(BCF)	3	5	(-0.56, 4.69)	$3.22^{ab} \pm 2.23$	14.2%		
	S2.4	Aqueous with uncontaminated food	Aqueous with uncontaminated food	Chronic	log(BCF)	4	28	(2.55, 3.78)	$3.19^{ab} \pm 0.29$	NA		
	S4.2	Aqueous and dietary	Clean water	Acute	log(BAF)	1	7	(2.31, 4.05)	$3.30^{ab} \pm 0.66$	NA		
	S4.4	Aqueous and dietary	NA	Chronic	log(BAF)	1	6	(2.97, 3.92)	$3.49^{ab} \pm 0.38$	NA		
Fish	S1.1	Aqueous	NA	Acute	log(BCF)	2	3	(0.02, 2.51)	$0.93^{a} \pm 1.37$	NA		
	S1.2	Aqueous	Clean water	Acute	log(BCF)	2	3	(0.30, 3.59)	$2.21^{ab} \pm 1.71$	47.0%		
	S2.1	Aqueous with uncontaminated food	Not reported	Acute	log(BCF)	4	4	(0.15, 2.64)	$1.58^{ab} \pm 1.10$	NA		
	S2.2	Aqueous with uncontaminated food	Clean water	Acute	log(BCF)	1	1		2.33	NA	(0.02, 3.79)	$2.05^{b} \pm 1.28$
	S2.3	Aqueous with uncontaminated food	Clean water and food	Acute	log(BCF)	1	2	(1.40, 2.25)	$1.82^{ab} \pm 0.60$	0.0%		
	S4.1	Aqueous and dietary	Not reported	Acute	log(BAF)	1	3	(0.11, 1.55)	$0.98^{a} \pm 0.76$	NA		
	S4.4	Aqueous and dietary	NA	Chronic	log(BAF)	1	6	(3.04, 3.79)	$3.40^{b} \pm 0.32$	NA		
Zooplankton	S3.1	Dietary	NA	Acute	BMF	6	22	(0.01, 122)	$25.83^{a} \pm 35.46$	NA	(0.01, 122)	$17.38^{a} \pm 28.62$
	S3.2	Dietary	Clean water	Acute	BMF	3	29	(0.05, 87.36)	$11.29^{a} \pm 21.23$	NA		
Fish	S3.1	Dietary	NA	Acute	BMF	5	5	(0.001, 0.67)	$0.14^{a} \pm 0.26$	NA	(0.001, 0.67)	$0.13^{b} \pm 0.22$
	S3.3	Dietary	Clean water and food	Acute	BMF	1	2	(0.06, 0.18)	$0.12^{a} \pm 0.09$	51%		

Table S3: Wet-to-dry weight ratio for phytoplankton, zooplankton and fish to convert the wet weight body burden to the dry weight body burden. When there are multiple data sources for the same target tissue, the average conversion ratio was taken.

**Organisms**	**Whole body/ tissue**	**Conversion ratio**	**References**
Phytoplankton	Whole body	10	(Chen et al., 2016)
Zooplankton	Whole body	12.5	(Tervonen et al., 2010)
Fish	Liver	3.52	(Kalay, 2000)
Fish	Liver	3.97	(Nimmo et al., 2016)
Fish	Gills	4.1	(Kalay, 2000)
Fish	Muscle	3.09	(Kalay, 2000)
Fish	Muscle	3.51	(Abdallah, 2008)
Fish	Whole body/ other organs	5	(Avenant-Oldewage & Marx, 2000)

Table S4: Exposure time (days) thresholds distinguishing acute studies and chronic studies (Wigger & Nowack, 2019).

	**Acute**	**Chronic**	**References**
Phytoplankton	<3	≥3	(OECD, 2011)
Zooplankton	<21	≥21	(OECD, 2012)
Fish	<28	≥28	(OECD, 2009)

Table S5: Organisms in different functional feeding groups of zooplankton.

Functional feeding group	Species full name	Species common name
Filter feeder	Ceriodaphnia dubia	Water Flea
Filter feeder	Daphnia magna	Water Flea
Filter feeder	Cyclops sp.	Copepod
Shredder	Gammarus fossarum	-
Gathering collector	Tetrahymena thermophila	-
Scraper	Lymnea stagnalis	Great pond snail
Scraper	Thamnocephalus platyurus	-
Scraper	Biomphalaria glabrata	Freshwater snail
Scraper	Cipangopaludina cathayensis	Freshwater snail

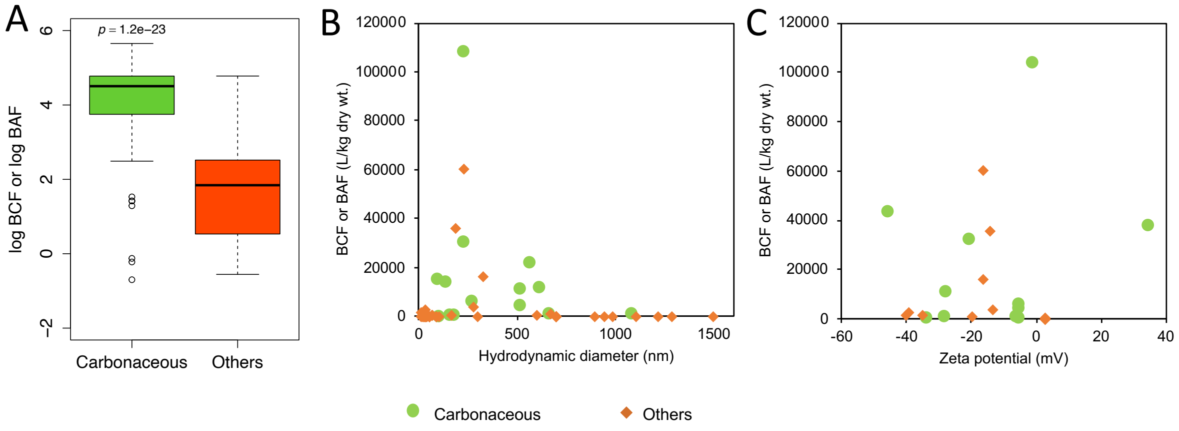

Figure S4: (A) Comparison of bioaccumulation level of carbonaceous ENMs (n = 99) and other ENMs (n = 78) (TiO_2 excluded), where n represents the number of data points. (B) Influence of the hydrodynamic diameter and (C) the zeta potential on the bioaccumulation for carbonaceous ENMs and other ENMs (TiO_2 excluded) respectively.

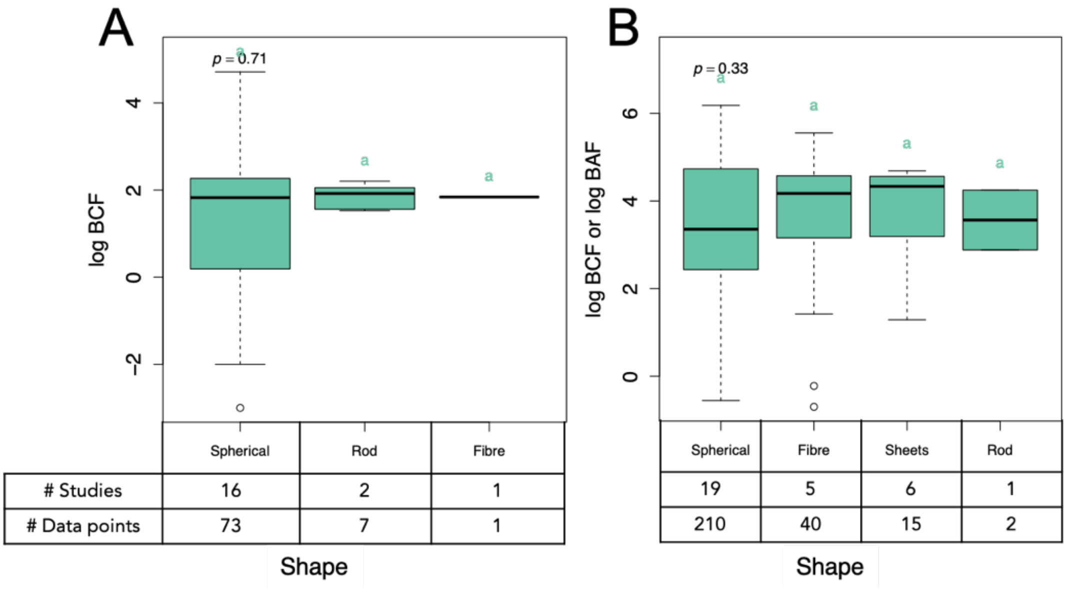

Figure S5: Bioaccumulation level of different shapes of ENMs in (A) phytoplankton and (B) zooplankton.

Table S6: List of references used for building the ENM bioaccumulation database:

- Abd El-Atti, M., Desouky, M. M. A., Mohamadien, A., & Said, R. M. (2019). Effects of titanium dioxide nanoparticles on red swamp crayfish, Procambarus clarkii: Bioaccumulation, oxidative stress and histopathological biomarkers. EGYPTIAN JOURNAL OF AQUATIC RESEARCH, 45(1), 11–18. https://doi.org/10.1016/j.ejar.2019.01.001
- Asztemborska, M., Jakubiak, M., Steborowski, R., Chajduk, E., & Bystrzejewska-Piotrowska, G. (2018). Titanium Dioxide Nanoparticle Circulation in an Aquatic Ecosystem. WATER AIR AND SOIL POLLUTION, 229(6). https://doi.org/10.1007/s11270-018-3852-8
- Ates, M., Demir, V., Adiguzel, R., & Arslan, Z. (2013). Bioaccumulation, Subacute Toxicity, and Tissue Distribution of Engineered Titanium Dioxide Nanoparticles in Goldfish (Carassius auratus). JOURNAL OF NANOMATERIALS, 2013. https://doi.org/10.1155/2013/460518
- Ates, M., Demir, V., Arslan, Z., Kaya, H., Yilmaz, S., & Camas, M. (2016). Chronic exposure of tilapia (Oreochromis niloticus) to iron oxide nanoparticles: Effects of particle morphology on accumulation, elimination, hematology and immune responses. AQUATIC TOXICOLOGY, 177, 22–32. https://doi.org/10.1016/j.aquatox.2016.05.005
- Avellan, A., Simonin, M., McGivney, E., Bossa, N., Spielman-Sun, E., Rocca, J. D., Bernhardt, E. S., Geitner, N. K., Unrine, J. M., Wiesner, M. R., & Lowry V, G. (2018). Gold nanoparticle biodissolution by a freshwater macrophyte and its associated microbiome. NATURE NANOTECHNOLOGY, 13(11), 1072+. https://doi.org/10.1038/s41565-018-0231-y
- Baudrimont, M., Andrei, J., Mornet, S., Gonzalez, P., Mesmer-Dudons, N., Gourves, P.-Y., Jaffal, A., Dedourge-Geffard, O., Geffard, A., Geffard, O., Garric, J., & Feurtet-Mazel, A. (2018). Trophic transfer and effects of gold nanoparticles (AuNPs) in Gammarus fossarum from contaminated periphytic biofilm. ENVIRONMENTAL SCIENCE AND POLLUTION RESEARCH, 25(12, SI), 11181–11191. https://doi.org/10.1007/s11356-017-8400-3
- Caixeta, M. B., Araújo, P. S., Rodrigues, C. C., Gonçalves, B. B., Araújo, O. A., Bevilaqua, G. B., Malafaia, G., Silva, L. D., & Rocha, T. L. (2021). Risk assessment of iron oxide nanoparticles in an aquatic ecosystem: A case study on Biomphalaria glabrata. Journal of Hazardous Materials, 401. https://doi.org/10.1016/j.jhazmat.2020.123398
- Cano, A M, Maul, J. D., Saed, M., Irin, F., Shah, S. A., Green, M. J., French, A. D., Klein, D. M., Crago, J., & Canas-Carrell, J. E. (2018). Trophic Transfer and Accumulation of Multiwalled Carbon Nanotubes in the Presence of Copper Ions in Daphnia magna and Fathead Minnow (Pimephales promelas). Environmental Science and Technology, 52(2), 794–800. https://doi.org/10.1021/acs.est.7b03522
- Cano, Amanda M, Maul, J. D., Saed, M., Shah, S. A., Green, M. J., & Canas-Carrell, J. E. (2017). BIOACCUMULATION, STRESS, AND SWIMMING IMPAIRMENT IN DAPHNIA MAGNA EXPOSED TO MULTIWALLED CARBON NANOTUBES, GRAPHENE, AND GRAPHENE OXIDE. ENVIRONMENTAL TOXICOLOGY AND CHEMISTRY, 36(8), 2199–2204. https://doi.org/10.1002/etc.3754
- Carmo, T. L. L., Azevedo, V. C., Siqueira, P. R., Galvao, T. D., Santos, F. A., Martinez, C. B. R., Appoloni, C. R., & Fernandes, M. N. (2018). Mitochondria-rich cells adjustments and ionic balance in the Neotropical fish Prochilodus lineatus exposed to titanium dioxide nanoparticles. AQUATIC TOXICOLOGY, 200, 168–177. https://doi.org/10.1016/j.aquatox.2018.05.006
- Chen, J., Li, H., Han, X., & Wei, X. (2015). Transmission and Accumulation of Nano-TiO2 in a 2-Step Food Chain (Scenedesmus obliquus to Daphnia magna). Bulletin of Environmental Contamination and Toxicology, 95(2), 145–149. https://doi.org/10.1007/s00128-015-1580-y
- Chen, Q, Hu, X., Yin, D., & Wang, R. (2016). Effect of subcellular distribution on nC60 uptake and transfer efficiency from Scenedesmus obliquus to Daphnia magna. Ecotoxicology and Environmental Safety, 128, 213–221. https://doi.org/10.1016/j.ecoenv.2016.02.026
- Chen, Qiqing, Yin, D., Li, J., & Hu, X. (2014). THE EFFECTS OF HUMIC ACID ON THE UPTAKE AND DEPURATION OF FULLERENE AQUEOUS SUSPENSIONS IN TWO AQUATIC ORGANISMS. ENVIRONMENTAL TOXICOLOGY AND CHEMISTRY, 33(5), 1090–1097. https://doi.org/10.1002/etc.2539
- Chen, X., Zhu, Y., Yang, K., Zhu, L., & Lin, D. (2019). Nanoparticle TiO2 size and rutile content impact bioconcentration and biomagnification from algae to daphnia. Environmental Pollution, 247, 421–430. https://doi.org/10.1016/j.envpol.2019.01.022
- Clemente, Z., Castro, V. L., Feitosa, L. O., Lima, R., Jonsson, C. M., Maia, A. H. N., & Fraceto, L. F. (2013). Fish exposure to nano-TiO2 under different experimental conditions: Methodological aspects for nanoecotoxicology investigations. SCIENCE OF THE TOTAL ENVIRONMENT, 463, 647–656. https://doi.org/10.1016/j.scitotenv.2013.06.022
- Cunha, C., Silva, L., Paulo, J., Faria, M., Nogueira, N., & Cordeiro, N. (2020). Microalgal-based biopolymer for nano- and microplastic removal: a possible biosolution for wastewater treatment. Environmental Pollution, 263. https://doi.org/10.1016/j.envpol.2020.114385
- Dalai, S., Iswarya, V., Bhuvaneshwari, M., Pakrashi, S., Chandrasekaran, N., & Mukherjee, A. (2014). Different modes of TiO2 uptake by Ceriodaphnia dubia: Relevance to toxicity and bioaccumulation. AQUATIC TOXICOLOGY, 152, 139–146. https://doi.org/10.1016/j.aquatox.2014.04.002

- Dalai, S., Pakrashi, S., Chandrasekaran, N., & Mukherjee, A. (2013). Acute Toxicity of TiO2 Nanoparticles to Ceriodaphnia dubia under Visible Light and Dark Conditions in a Freshwater System. PLOS ONE, 8(4). https://doi.org/10.1371/journal.pone.0062970
- Ding, J., Huang, Y., Liu, S., Zhang, S., Zou, H., Wang, Z., Zhu, W., & Geng, J. (2020). Toxicological effects of nano- and micro-polystyrene plastics on red tilapia: Are larger plastic particles more harmless? JOURNAL OF HAZARDOUS MATERIALS, 396. https://doi.org/10.1016/j.jhazmat.2020.122693
- Dong, S., Xia, T., Yang, Y., Lin, S., & Mao, L. (2018). Bioaccumulation of C-14-Labeled Graphene in an Aquatic Food Chain through Direct Uptake or Trophic Transfer. ENVIRONMENTAL SCIENCE & TECHNOLOGY, 52(2), 541–549. https://doi.org/10.1021/acs.est.7b04339
- Fan, W., Liu, Y., Xu, Z., Wang, X., Li, X., & Luo, S. (2016). The mechanism of chronic toxicity to: Daphnia magna induced by graphene suspended in a water column. Environmental Science: Nano, 3(6), 1405–1415. https://doi.org/10.1039/c6en00361c
- Federici, G., Shaw, B. J., & Handy, R. D. (2007). Toxicity of titanium dioxide nanoparticles to rainbow trout (Oncorhynchus mykiss): Gill injury, oxidative stress, and other physiological effects. AQUATIC TOXICOLOGY, 84(4), 415–430. https://doi.org/10.1016/j.aquatox.2007.07.009
- Feng, Y., Lu, K., Mao, L., Guo, X., Gao, S., & Petersen, E. J. (2015). Degradation of C-14-labeled few layer graphene via Fenton reaction: Reaction rates, characterization of reaction products, and potential ecological effects. WATER RESEARCH, 84, 49–57. https://doi.org/10.1016/j.watres.2015.07.016
- Fouqueray, M., Noury, P., Dherret, L., Chaurand, P., Abbaci, K., Labille, J., Rose, J., & Garric, J. (2013a). Exposure of juvenile Danio rerio to aged TiO2 nanomaterial from sunscreen. Environmental Science and Pollution Research, 20(5), 3340–3350. https://doi.org/10.1007/s11356-012-1256-7
- Fouqueray, M., Noury, P., Dherret, L., Chaurand, P., Abbaci, K., Labille, J., Rose, J., & Garric, J. (2013b). Exposure of juvenile Danio rerio to aged TiO_2 nanomaterial from sunscreen. Environmental Science and Pollution Research International, 20(5), 3340–3350. https://doi.org/10.1007/s11356-012-1256-7
- Geffroy, B., Ladhar, C., Cambier, S., Treguer-Delapierre, M., Brethes, D., & Bourdineaud, J.-P. (2012). Impact of dietary gold nanoparticles in zebrafish at very low contamination pressure: The role of size, concentration and exposure time. NANOTOXICOLOGY, 6(2), 144–160. https://doi.org/10.3109/17435390.2011.562328
- Glenn, J. B., & Klaine, S. J. (2013). Abiotic and biotic factors that influence the bioavailability of gold nanoparticles to aquatic macrophytes. Environmental Science and Technology, 47(18), 10223–10230. https://doi.org/10.1021/es4020508
- Gray, E. P., Coleman, J. G., Bednar, A. J., Kennedy, A. J., Ranville, J. F., & Higgins, C. P. (2013). Extraction and Analysis of Silver and Gold Nanoparticles from Biological Tissues Using Single Particle Inductively Coupled Plasma Mass Spectrometry. ENVIRONMENTAL SCIENCE & TECHNOLOGY, 47(24), 14315–14323. https://doi.org/10.1021/es403558c
- Guimarães, A. T. B., Estrela, F. N., Rodrigues, A. S. D. L., Chagas, T. Q., Pereira, P. S., Silva, F. G., & Malafaia, G. (2021). Nanopolystyrene particles at environmentally relevant concentrations causes behavioral and biochemical changes in juvenile grass carp (Ctenopharyngodon idella). Journal of Hazardous Materials, 403. https://doi.org/10.1016/j.jhazmat.2020.123864
- Guo, X., Dong, S., Petersen, E. J., Gao, S., Huang, Q., & Mao, L. (2013). Biological Uptake and Depuration of Radio-labeled Graphene by Daphnia magna. ENVIRONMENTAL SCIENCE & TECHNOLOGY, 47(21), 12524–12531. https://doi.org/10.1021/es403230u
- Hu, J., Wang, D., Wang, J., & Wang, J. (2012). Bioaccumulation of Fe2O3(magnetic) nanoparticles in Ceriodaphnia dubia. ENVIRONMENTAL POLLUTION, 162, 216–222. https://doi.org/10.1016/j.envpol.2011.11.016
- Hudson, M. L., Costello, D. M., Daley, J. M., & Burton, G. A. (2019). Species-Specific (Hyalella azteca and Lymnea stagnalis) Dietary Accumulation of Gold Nano-particles Associated with Periphyton. BULLETIN OF ENVIRONMENTAL CONTAMINATION AND TOXICOLOGY, 103(2), 255–260. https://doi.org/10.1007/s00128-019-02620-2
- Hull, M. S., Chaurand, P., Rose, J., Auffan, M., Bottero, J. Y., Jones, J. C., Schultz, I. R., & Vikesland, P. J. (2011). Filter-feeding bivalves store and biodeposit colloidally stable gold nanoparticles. Environmental Science and Technology, 45(15), 6592–6599. https://doi.org/10.1021/es200809c
- Iswarya, V., Bhuvaneshwari, M., Chandrasekaran, N., & Mukherjee, A. (2018). Trophic transfer potential of two different crystalline phases of TiO2 NPs from Chlorella sp to Ceriodaphnia dubia. AQUATIC TOXICOLOGY, 197, 89–97. https://doi.org/10.1016/j.aquatox.2018.02.003
- Johnston, B. D., Scown, T. M., Moger, J., Cumberland, S. A., Baalousha, M., Linge, K., van Aerle, R., Jarvis, K., Lead, J. R., & Tyler, C. R. (2010). Bioavailability of Nanoscale Metal Oxides TiO2, CeO2, and ZnO to Fish. ENVIRONMENTAL SCIENCE & TECHNOLOGY, 44(3), 1144–1151. https://doi.org/10.1021/es901971a
- Krystek, P., Brandsma, S., Leonards, P., & de Boer, J. (2016). Exploring methods for compositional and particle size analysis of noble metal nanoparticles in Daphnia magna. TALANTA, 147, 289–295. https://doi.org/10.1016/j.talanta.2015.09.063
- Lee, W.-M., Yoon, S.-J., Shin, Y.-J., & An, Y.-J. (2015). Trophic transfer of gold nanoparticles from Euglena gracilis or Chlamydomonas reinhardtii to Daphnia magna. Environmental Pollution, 201, 10–16. https://doi.org/10.1016/j.envpol.2015.02.021
- Liu, Y., Jin, W., Zhou, X., Han, S.-F., Tu, R., Feng, X., Jensen, P. D., & Wang, Q. (2019). Efficient harvesting of Chlorella pyrenoidosa and Scenedesmus obliquus cultivated in urban sewage by magnetic flocculation using nano-Fe3O4 coated with polyethyleneimine. Bioresource Technology, 290. https://doi.org/10.1016/j.biortech.2019.121771

- Lu, H., Fan, W., Dong, H., & Liu, L. (2017). Dependence of the irradiation conditions and crystalline phases of TiO2 nanoparticles on their toxicity to Daphnia magna. ENVIRONMENTAL SCIENCE-NANO, 4(2), 406–414. https://doi.org/10.1039/c6en00391e
- Lu, K., Dong, S., Petersen, E. J., Niu, J., Chang, X., Wang, P., Lin, S., Gao, S., & Mao, L. (2017). Biological Uptake, Distribution, and Depuration of Radio-Labeled Graphene in Adult Zebrafish: Effects of Graphene Size and Natural Organic Matter. ACS NANO, 11(3), 2872–2885. https://doi.org/10.1021/acsnano.6b07982
- Lv, X., Huang, B., Zhu, X., Jiang, Y., Chen, B., Tao, Y., Zhou, J., & Cai, Z. (2017). Mechanisms underlying the acute toxicity of fullerene to Daphnia magna: Energy acquisition restriction and oxidative stress. WATER RESEARCH, 123, 696–703. https://doi.org/10.1016/j.watres.2017.07.023
- Lv, X., Yang, Y., Tao, Y., Jiang, Y., Chen, B., Zhu, X., Cai, Z., & Li, B. (2018). A mechanism study on toxicity of graphene oxide to Daphnia magna: Direct link between bioaccumulation and oxidative stress. ENVIRONMENTAL POLLUTION, 234, 953–959. https://doi.org/10.1016/j.envpol.2017.12.034
- Maes, H. M., Stibany, F., Giefers, S., Daniels, B., Deutschmann, B., Baumgartner, W., & Schaeffer, A. (2014). Accumulation and Distribution of Multiwalled Carbon Nanotubes in Zebrafish (Danio rerio). ENVIRONMENTAL SCIENCE & TECHNOLOGY, 48(20), 12256–12264. https://doi.org/10.1021/es503006v
- Malejko, J., Szymańska, N., Bajguz, A., & Godlewska-Żyłkiewicz, B. (2019). Studies on the uptake and transformation of gold(iii) and gold nanoparticles in a water-green algae environment using mass spectrometry techniques. Journal of Analytical Atomic Spectrometry, 34(7), 1485–1496. https://doi.org/10.1039/c9ja00132h
- Matouke, M. M., & Mustapha, M. (2018). Bioaccumulation and physiological effects of copepods sp (Eucyclop sp.) fed Chlorella ellipsoides exposed to titanium dioxide (TiO2) nanoparticles and lead (Pb2+). AQUATIC TOXICOLOGY, 198, 30–39. https://doi.org/10.1016/j.aquatox.2018.02.013
- Mehennaoui, K., Cambier, S., Serchi, T., Ziebel, J., Lentzen, E., Valle, N., Guerold, F., Thomann, J.-S., Giamberini, L., & Gutleb, A. C. (2018). Do the pristine physico-chemical properties of silver and gold nanoparticles influence uptake and molecular effects on Gammarus fossarum (Crustacea Amphipoda)? SCIENCE OF THE TOTAL ENVIRONMENT, 643, 1200–1215. https://doi.org/10.1016/j.scitotenv.2018.06.208
- Morgalev, Y. N., Khoch, N. S., Morgaleva, T. G., Gulik, E. S., Borilo, G. A., Bulatova, U. A., Morgalev, S. Y., & Ponyavina, E. V. (2010). Biotesting Nanomaterials: Transmissibility of Nanoparticles into a Food Chain. NANOTECHNOLOGIES IN RUSSIA, 5(11–12), 851–856. https://doi.org/10.1134/S1995078010110157
- Mortimer, M., Petersen, E. J., Buchholz, B. A., Orias, E., & Holden, P. A. (2016). Bioaccumulation of Multiwall Carbon Nanotubes in Tetrahymena thermophila by Direct Feeding or Trophic Transfer. ENVIRONMENTAL SCIENCE & TECHNOLOGY, 50(16), 8876–8885. https://doi.org/10.1021/acs.est.6b01916
- Omidzahir, S., Bayi, M. A., Kardel, F., & Mazandarani, M. (2019). Effects of iron oxide nano-particles on the intestinal tissue of common carp, Cyprinus Carpio. Iranian Journal of Toxicology, 13(3), 33–38. https://www.scopus.com/inward/record.uri?eid=2-s2.0-85083885365&partnerID=40&md5=d835f14ff6b156166a136f60c97ae151
- Pakarinen, K., Petersen, E. J., Alvila, L., Waissi-Leinonen, G. C., Akkanen, J., Leppanen, M. T., & Kukkonen, J. V. K. (2013). A screening study on the fate of fullerenes (nC60) and their toxic implications in natural freshwaters. ENVIRONMENTAL TOXICOLOGY AND CHEMISTRY, 32(6), 1224–1232. https://doi.org/10.1002/etc.2175
- Pakrashi, S, Dalai, S., Chandrasekaran, N., & Mukherjee, A. (2014). Trophic transfer potential of aluminium oxide nanoparticles using representative primary producer (Chlorella ellipsoides) and a primary consumer (Ceriodaphnia dubia). Aquatic Toxicology, 152, 74–81. https://doi.org/10.1016/j.aquatox.2014.03.024
- Pakrashi, Sunandan, Dalai, S., Humayun, A., Chakravarty, S., Chandrasekaran, N., & Mukherjee, A. (2013). Ceriodaphnia dubia as a Potential Bio-Indicator for Assessing Acute Aluminum Oxide Nanoparticle Toxicity in Fresh Water Environment. PLOS ONE, 8(9). https://doi.org/10.1371/journal.pone.0074003
- Park, H.-G., & Yeo, M.-K. (2013). Effects of TiO2 nanoparticles and nanotubes on zebrafish caudal fin regeneration. MOLECULAR & CELLULAR TOXICOLOGY, 9(4), 375–383. https://doi.org/10.1007/s13273-013-0046-8
- Patra, M., Ma, X., Isaacson, C., Bouchard, D., Poynton, H., Lazorchak, J. M., & Rogers, K. R. (2011). CHANGES IN AGGLOMERATION OF FULLERENES DURING INGESTION AND EXCRETION IN THAMNOCEPHALUS PLATYURUS. ENVIRONMENTAL TOXICOLOGY AND CHEMISTRY, 30(4), 828–835. https://doi.org/10.1002/etc.468
- Perrier, F., Baudrimont, M., Mornet, S., Mesmer-Dudons, N., Lacomme, S., Etcheverria, B., Simon, O., & Feurtet-Mazel, A. (2018). Gold nanoparticle trophic transfer from natural biofilm to grazer fish. GOLD BULLETIN, 51(4), 163–173. https://doi.org/10.1007/s13404-018-0241-4
- Petersen, E. J., Akkanen, J., Kukkonen, J. V. K., & Weber, W. J. (2009). Biological uptake and depuration of carbon nanotubes by daphnia magna. Environmental Science and Technology, 43(8), 2969–2975. https://doi.org/10.1021/es8029363
- Petersen, E. J., Pinto, R. A., Mai, D. J., Landrum, P. F., & Weber Jr., W. J. (2011). Influence of Polyethyleneimine Graftings of Multi-Walled Carbon Nanotubes on their Accumulation and Elimination by and Toxicity to Daphnia magna. ENVIRONMENTAL SCIENCE & TECHNOLOGY, 45(3), 1133–1138. https://doi.org/10.1021/es1030239
- Potouridis, T., Voelker, J., Alsenz, H., Oetken, M., & Puettmann, W. (2014). Using ICP-qMS to trace the uptake of nanoscale titanium dioxide by microalgae-potential disadvantages of vegetable reference material. ANALYTICAL AND BIOANALYTICAL CHEMISTRY, 406(11), 2495–2502. https://doi.org/10.1007/s00216-014-7666-2
- Ramsden, C. S., Smith, T. J., Shaw, B. J., & Handy, R. D. (2009). Dietary exposure to titanium dioxide nanoparticles in rainbow trout, (Oncorhynchus mykiss): no effect on growth, but subtle biochemical disturbances in the brain. ECOTOXICOLOGY, 18(7), 939–951. https://doi.org/10.1007/s10646-009-0357-7

- Rhiem, S., Riding, M. J., Baumgartner, W., Martin, F. L., Semple, K. T., Jones, K. C., Schäffer, A., & Maes, H. M. (2015). Interactions of multiwalled carbon nanotubes with algal cells: Quantification of association, visualization of uptake, and measurement of alterations in the composition of cells. Environmental Pollution, 196, 431–439. https://doi.org/10.1016/j.envpol.2014.11.011
- Rist, S., Baun, A., & Hartmann, N. B. (2017). Ingestion of micro- and nanoplastics in Daphnia magna – Quantification of body burdens and assessment of feeding rates and reproduction. Environmental Pollution, 228, 398–407. https://doi.org/10.1016/j.envpol.2017.05.048
- Rosenkranz, P., Chaudhry, Q., Stone, V., & Fernandes, T. F. (2009). A COMPARISON OF NANOPARTICLE AND FINE PARTICLE UPTAKE BY DAPHNIA MAGNA. ENVIRONMENTAL TOXICOLOGY AND CHEMISTRY, 28(10), 2142–2149. https://doi.org/10.1897/08-559.1
- Sayadi, M. H., Mansouri, B., Shahri, E., Tyler, C. R., Shekari, H., & Kharkan, J. (2020). Exposure effects of iron oxide nanoparticles and iron salts in blackfish (Capoeta fusca): Acute toxicity, bioaccumulation, depuration, and tissue histopathology. CHEMOSPHERE, 247. https://doi.org/10.1016/j.chemosphere.2020.125900
- Sendra, M., Sánchez-Quiles, D., Blasco, J., Moreno-Garrido, I., Lubián, L. M., Pérez-García, S., & Tovar-Sánchez, A. (2017). Effects of TiO2nanoparticles and sunscreens on coastal marine microalgae: Ultraviolet radiation is key variable for toxicity assessment. Environment International, 98, 62–68. https://doi.org/10.1016/j.envint.2016.09.024
- Shahzad, K., Khan, M. N., Jabeen, F., Kosour, N., Sohail, M., Khan, M. K. A., & Ahmad, M. (2017). Bioaccumulation of manufactured titanium dioxide (TiO2), copper oxide (CuO) and zinc oxides (ZnO) nanoparticles in the soft tissues of tilapia (Oreochromis mossambicus). Punjab University Journal of Zoology, 32(2), 237–243. https://www.scopus.com/inward/record.uri?eid=2-s2.0-85048087864&partnerID=40&md5=30391c55ccebf81cd6ac01b99564d06d
- Shi, X., Li, Z., Chen, W., Qiang, L., Xia, J., Chen, M., Zhu, L., & Alvarez, P. J. J. (2016). Fate of TiO2 nanoparticles entering sewage treatment plants and bioaccumulation in fish in the receiving streams. NanoImpact, 3–4, 96–103. https://doi.org/10.1016/j.impact.2016.09.002
- Skjolding, L. M., Asmonaite, G., Jolck, R. I., Andresen, T. L., Selck, H., Baun, A., & Sturve, J. (2017). An assessment of the importance of exposure routes to the uptake and internal localisation of fluorescent nanoparticles in zebrafish (Danio rerio), using light sheet microscopy. NANOTOXICOLOGY, 11(3), 351–359. https://doi.org/10.1080/17435390.2017.1306128
- Skjolding, L. M., Kern, K., Hjorth, R., Hartmann, N., Overgaard, S., Ma, G., Veinot, J. G. C., & Baun, A. (2014). Uptake and depuration of gold nanoparticles in Daphnia magna. Ecotoxicology, 23(7), 1172–1183. https://doi.org/10.1007/s10646-014-1259-x
- Su, Y., Tong, X., Huang, C., Chen, J., Liu, S., Gao, S., Mao, L., & Xing, B. (2018). Green Algae as Carriers Enhance the Bioavailability of 14C-Labeled Few-Layer Graphene to Freshwater Snails. Environmental Science and Technology, 52(3), 1591–1601. https://doi.org/10.1021/acs.est.7b05796
- Suganya, D., Ramakritinan, C. M., & Rajan, M. R. (2018). Adverse Effects of Genotoxicity, Bioaccumulation and Ionoregulatory Modulation of Two Differently Synthesized Iron Oxide Nanoparticles on Zebrafish (Danio rerio). JOURNAL OF INORGANIC AND ORGANOMETALLIC POLYMERS AND MATERIALS, 28(6), 2603–2611. https://doi.org/10.1007/s10904-018-0935-3
- Sung, H. K., Jo, E., Kim, E., Yoo, S., Lee, J., Kim, P., Kim, Y., & Eom, I.-C. (2018). Analysis of gold and silver nanoparticles internalized by zebrafish (Danio rerio) using single particle-inductively coupled plasma-mass spectrometry. CHEMOSPHERE, 209, 815–822. https://doi.org/10.1016/j.chemosphere.2018.06.149
- Tao, X., He, Y., Zhang, B., Chen, Y., & Hughes, J. B. (2011). Effects of stable aqueous fullerene nanocrystal (nC(60)) on Daphnia magna: Evaluation of hop frequency and accumulations under different conditions. JOURNAL OF ENVIRONMENTAL SCIENCES, 23(2), 322–329. https://doi.org/10.1016/S1001-0742(10)60409-3
- Tervonen, K., Waissi, G., Petersen, E. J., Akkanen, J., & Kukkonen, J. V. K. (2010). ANALYSIS OF FULLERENE-C-60 AND KINETIC MEASUREMENTS FOR ITS ACCUMULATION AND DEPURATION IN DAPHNIA MAGNA. ENVIRONMENTAL TOXICOLOGY AND CHEMISTRY, 29(5), 1072–1078. https://doi.org/10.1002/etc.124
- Uzo-God, O. C., Agarwal, A., & Singh, N. B. (2019). Effects of dietary nano and macro iron oxide (Fe2O3) on the growth, biochemical, and hematological profiles of African catfish (Clarias gariepinus) fingerlings. Journal of Applied Aquaculture, 31(2), 153–171. https://doi.org/10.1080/10454438.2018.1534704
- Wang, D., Hu, J., Forthaus, B. E., & Wang, J. (2011). Synergistic toxic effect of nano-Al2O3 and As(V) on Ceriodaphnia dubia. ENVIRONMENTAL POLLUTION, 159(10), 3003–3008. https://doi.org/10.1016/j.envpol.2011.04.019
- Wray, A. T., & Klaine, S. J. (2015). MODELING THE INFLUENCE OF PHYSICOCHEMICAL PROPERTIES ON GOLD NANOPARTICLE UPTAKE AND ELIMINATION BY DAPHNIA MAGNA. ENVIRONMENTAL TOXICOLOGY AND CHEMISTRY, 34(4), 860–872. https://doi.org/10.1002/etc.2881
- Wright, M. V, Matson, C. W., Baker, L. F., Castellon, B. T., Watkins, P. S., & King, R. S. (2018). Titanium dioxide nanoparticle exposure reduces algal biomass and alters algal assemblage composition in wastewater effluent-dominated stream mesocosms. The Science of the Total Environment, 626, 357–365. https://doi.org/10.1016/j.scitotenv.2018.01.050
- Xiao, B., Zhang, Y., Wang, X., Chen, M., Sun, B., Zhang, T., & Zhu, L. (2019). Occurrence and trophic transfer of nanoparticulate Ag and Ti in the natural aquatic food web of Taihu Lake, China. ENVIRONMENTAL SCIENCE-NANO, 6(11), 3431–3441. https://doi.org/10.1039/c9en00797k
- Yeo, M.-K., & Nam, D.-H. (2013). Influence of different types of nanomaterials on their bioaccumulation in a paddy microcosm: A comparison of TiO2 nanoparticles and nanotubes. ENVIRONMENTAL POLLUTION, 178, 166–172. https://doi.org/10.1016/j.envpol.2013.03.040

- Zeumer, R., Galhano, V., Monteiro, M. S., Kuehr, S., Knopf, B., Meisterjahn, B., Soares, A. M. V. M., Loureiro, S., Lopes, I., & Schlechtriem, C. (2020). Chronic effects of wastewater-borne silver and titanium dioxide nanoparticles on the rainbow trout (Oncorhynchus mykiss). SCIENCE OF THE TOTAL ENVIRONMENT, 723. https://doi.org/10.1016/j.scitotenv.2020.137974
- Zhu, X., Chang, Y., & Chen, Y. (2010). Toxicity and bioaccumulation of TiO_2 nanoparticle aggregates in Daphnia magna. Chemosphere, 78(3), 209–215. https://doi.org/10.1016/j.chemosphere.2009.11.013
- Zhu, X., Wang, J., Zhang, X., Chang, Y., & Chen, Y. (2010). Trophic transfer of TiO_2 nanoparticles from daphnia to zebrafish in a simplified freshwater food chain. Chemosphere, 79(9), 928–933. https://doi.org/10.1016/j.chemosphere.2010.03.022

References

Abdallah, M. A. M. (2008). Trace element levels in some commercially valuable fish species from coastal waters of Mediterranean Sea, Egypt. *Journal of Marine Systems*, *73*(1–2), 114–122. https://doi.org/10.1016/j.jmarsys.2007.09.006

Avenant-Oldewage, A., & Marx, H. (2000). Manganese, nickel and strontium bioaccumulation in the tissues of the African sharptooth catfish, Clarias gariepinus from the Olifants River, Kruger National Park. *Koedoe*, *43*(2), 17–33. https://doi.org/10.4102/koedoe.v43i2.196

Chen, Q., Hu, X., Yin, D., & Wang, R. (2016). Effect of subcellular distribution on nC60 uptake and transfer efficiency from Scenedesmus obliquus to Daphnia magna. *Ecotoxicology and Environmental Safety*, *128*, 213–221. https://doi.org/10.1016/j.ecoenv.2016.02.026

Kalay, M. (2000). Elimination of Essential (Cu, Zn) and Non-Essential (Cd, Pb) Metals from Tissues of a Freshwater Fish Tilapia zilli. *Turkish Journal of Zoology*, *24*(4), 429-436–436.

Nimmo, D. R., Herrmann, S. J., Carsella, J. S., McGarvy, C. M., Foutz, H. P., Herrmann-Hoesing, L. M., Gregorich, J. M., Turner, J. A., & Vanden Heuvel, B. D. (2016). Mercury and selenium in fish of Fountain Creek, Colorado (USA): possible sources and implications. *SpringerPlus*, *5*(1). https://doi.org/10.1186/s40064-016-2088-6

OECD. (2009). *Test No. 230: 21-day Fish Assay*. https://doi.org/https://doi.org/https://doi.org/10.1787/9789264076228-en

OECD. (2011). *Test No. 201: Freshwater Alga and Cyanobacteria, Growth Inhibition Test*. https://doi.org/https://doi.org/https://doi.org/10.1787/9789264069923-en

OECD. (2012). *Test No. 211: Daphnia magna Reproduction Test*. https://doi.org/https://doi.org/https://doi.org/10.1787/9789264185203-en

Tervonen, K., Waissi, G., Petersen, E. J., Akkanen, J., & Kukkonen, J. V. K. (2010). ANALYSIS OF FULLERENE-C-60 AND KINETIC MEASUREMENTS FOR ITS ACCUMULATION AND DEPURATION IN DAPHNIA MAGNA. *ENVIRONMENTAL TOXICOLOGY AND CHEMISTRY*, *29*(5), 1072–1078. https://doi.org/10.1002/etc.124

Wigger, H., & Nowack, B. (2019). Material-specific properties applied to an environmental risk assessment of engineered nanomaterials–implications on grouping and read-across concepts. *Nanotoxicology*. https://doi.org/10.1080/17435390.2019.1568604
